# mTORC1 impedes osteoclast differentiation via calcineurin and NFATc1

**DOI:** 10.1038/s42003-018-0028-4

**Published:** 2018-04-05

**Authors:** HoangDinh Huynh, Yihong Wan

**Affiliations:** Department of Pharmacology, The University of Texas Southwestern Medical Center, Dallas, TX 75390 USA

## Abstract

Rapamycins are immunosuppressant and anti-cancer drugs that inhibit the kinase mTOR. Clinically, they often cause bone pain, bone necrosis, and high bone turnover, yet the mechanisms are unclear. Here we show that mTORC1 activity is high in osteoclast precursors but downregulated upon RANKL treatment. Loss-of-function genetic models reveal that while early Raptor deletion in hematopoietic stem cells blunts osteoclastogenesis due to compromised proliferation/survival, late Raptor deletion in osteoclast precursors instead augments osteoclastogenesis. Gain-of-function genetic models by TSC1 deletion in HSCs or osteoclast precursors cause constitutive mTORC1 activation, impairing osteoclastogenesis. Pharmacologically, rapamycin treatment at low but clinically relevant doses exacerbates osteoclast differentiation and bone resorption, leading to bone loss. Mechanistically, RANKL inactivates mTORC1 via calcineurin-mediated mTORC1 dephosphorylation, consequently activating NFATc1 by reducing mTORC1-mediated NFATc1 phosphorylation. These findings uncover biphasic roles of mTORC1 in osteoclastogenesis, dosage-dependent effects of rapamycin on bone, and a previously unrecognized calcineurin–mTORC1–NFATc1 phosphorylation-regulatory signaling cascade.

## Abstract (web summary)

HoangDinh Huynh and Yihong Wan investigate the role of the mTORC1 pathway during osteoclastogenesis and find that the cytokine RANKL inactivates mTORC1 via calcineurin-mediated dephosphorylation, leading to activation of NFATc1 by reducing its phosphorylation. These findings have implications for bone diseases and mTORC1/NFATc1 signaling.

## Introduction

Bone is a dynamic tissue that undergoes continuous remodeling throughout the lifespan via balancing bone resorption by osteoclasts and bone formation by osteoblasts. Osteoclasts are of hematopoietic lineage, whereas osteoblasts are of mesenchymal origin. The synchronization between bone resorption and bone formation maintains bone mass and preserves bone quality in adult skeleton. However, excessive osteoclastogenesis and bone resorption under pathological states results in skeletal disorders such as osteoporosis, rheumatoid arthritis, Paget’s disease, and lytic bone metastasis of cancer malignancies, representing a major health concern and socioeconomic burden. Macrophage colony-stimulating factor (M-CSF) and receptor activator of nuclear factor-kappa B ligand (RANKL) are two cytokines essential for osteoclastogenesis^1–4^. M-CSF promotes the proliferation and survival of osteoclast precursors through its receptor, M-CSFR. Upon binding to its receptor RANK, RANKL triggers various downstream mitogen-activated protein kinase (MAPK) signaling cascades, such as p38, c-Jun N-terminal kinase (JNK), and extracellular signal-regulated kinase (ERK) pathways, as well as activates multiple transcriptional factors, including nuclear factor-activated T cells c1 (NFATc1), NFκB and AP1^5,6^. However, our understanding of the signaling pathways that govern osteoclast differentiation is far from complete.

The NFAT gene family, which consists of five members^7^, was identified three decades ago. This family is known to play key roles in many biological processes such as immune cell activation, lymphoid development, heart valve formation, cardiac and skeletal muscle hypertrophy, organization of vascular system, and osteoclast differentiation^8–12^. The functions of NFAT1-4 proteins are crucially regulated by the calcium/calmodulin-dependent phosphatase calcineurin^8,9,13^. Dephosphorylating NFAT proteins at serine residues by activated calcineurin induces a conformational change that exposes their nuclear localization signal and facilitates their translocation from cytosol into the nucleus, where they regulate the transcription of target genes such as cathepsin K (CTSK)^14–16^. In particular, NFATc1 (also known as NFAT2) is not only required but also sufficient for osteoclastogenesis, as its overexpression in osteoclast precursors induces osteoclast differentiation in the absence of RANKL in vitro and in vivo^12,17^.

Given NFATc1 is a master transcription factor for osteoclast differentiation, its activity must be dynamically regulated and tightly controlled. Prolonged NFATc1 activation over time may cause excessive osteoclastogenesis and bone resorption, leading to bone loss in various skeletal disorders^18–25^. Therefore, better molecular characterization of NFATc1 regulation in osteoclast will be important for the understanding of bone diseases and the development of new anti-resorptive therapeutic. Several transcriptional negative regulators of NFATc1 have been reported in osteoclasts, such as interferon regulatory factor-8 (IRF-8), V-maf musculoaponeurotic fibrosarcoma oncogene homolog B (MafB), inhibitors of differentiation (Ids), and LIM homeobox 2 (Lhx2)^26–29^. Recent studies have also uncovered negative regulations of NFATc1 through post-translational modifications and post-transcriptional regulations, such as ubiquitination, methylation, deacetylation, and non-coding RNA miRNA-124^30–34^.

The mechanistic target of rapamycin (mTOR) is a serine/threonine kinase in the phosphoinositide 3-kinase (PI3K)-related kinase family that forms two distinct complexes. mTOR complex 1 (mTORC1) contains Raptor and is sensitive to rapamycin, whereas mTORC2 contains Rictor and is rapamycin-insensitive^35^. mTORC1 is recognized for its role in regulating multiple cellular processes that control organismal growth and homeostasis, including protein synthesis, autophagy, metabolism and lipogenesis. Increasingly, the function of mTORC1 in immunity and inflammation has also been recognized. As such, rapamycin has been FDA approved as immunosuppressant and cancer drugs. Despite the biological and clinical importance, there are only a few reports on how mTORC1 impacts osteoclastogenesis, including two recent studies presenting conflicting results without clear molecular mechanisms^36,37^. In the present study, we have investigated the roles of mTORC1 signaling in osteoclastogenesis using comprehensive and integrated strategies combining genetic and pharmacological mouse models, gain- and loss-of-function approaches, in vitro and in vivo. Our findings have also revealed the underlying molecular mechanisms for the clinically observed rapamycin-induced high bone turnover and bone loss. These studies provide a unifying explanation for the seemingly contradictory observations in current basic and clinical research. More fundamentally, we have identified a previously unrecognized calcineurin—mTORC1—NFATc1 phosphorylation and dephosphorylation signaling cascade, which may exert widespread impact on many fields including cancer and immunity.

## Results

### mTORC1 signaling subsides during osteoclast differentiation

**Fig. 1:**
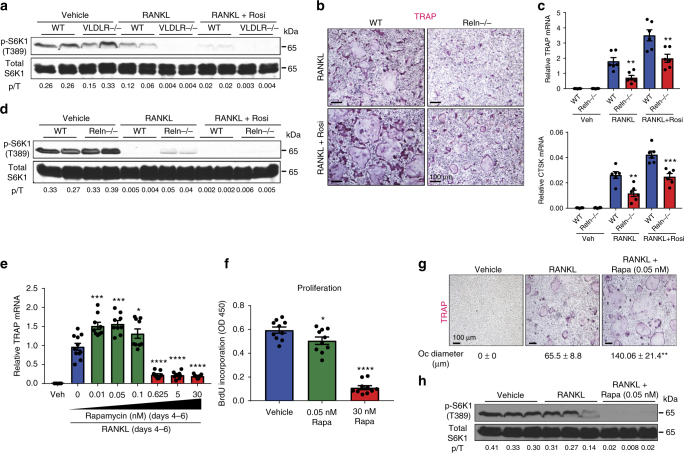
The inverse relationship of osteoclast differentiation and mTORC1 Signaling. **a** mTORC1 signaling in bone marrow osteoclast differentiation cultures from VLDLR−/− mice or wild-type (WT) littermate controls 60 h after RANKL treatment with or without rosiglitazone, measured by S6K1 phosphorylation. p/T, ratio of p-S6K1/total S6K1. **b** Representative images of TRAP-stained bone marrow osteoclast differentiation cultures from Reln−/− mice or WT littermate controls. **c** Expression of osteoclast markers in bone marrow osteoclast differentiation cultures (*n* = 6). **d** mTORC1 signaling in bone marrow osteoclast differentiation cultures from Reelin−/− mice or WT littermate controls 60 h after RANKL treatment with or without rosiglitazone, measured by S6K1 phosphorylation. p/T ratio of p-S6K1/total S6K1. **e** TRAP expression on day 6 of bone marrow osteoclast differentiation cultures treated with rapamycin at indicated dose and time (*n* = 9–11). **f** Osteoclast precursor proliferation by BrdU incorporation (*n* = 10). **g** Representative images of TRAP-stained osteoclast differentiation cultures on day 9. Quantification of osteoclast diameter (*n* = 18–20). ** compares RANKL + Rapa with RANKL. **h** mTORC1 signaling in bone marrow osteoclast differentiation cultures 40 h after RANKL treatment with or without rapamycin, measured by S6K1 phosphorylation. p/T ratio of p-S6K1/total S6K1. Error bars, SEM; *p < 0.05; **p < 0.01; ***p < 0.005; ****p < 0.001; n.s. non-significant. Full-size scans of immunoblots are shown in Supplementary Fig. 6

In the course of studying osteoclastogenesis, we found that there was an inverse relationship of osteoclast differentiation and mTORC1 signaling. Quantified by S6K1 phosphorylation, mTORC1 activity in osteoclast differentiation cultures was reduced by RANKL and further decreased by rosiglitazone, an agonist for the pro-osteoclastogenic nuclear receptor PPARγ^38^ (Fig. 1a, d). Moreover, mouse genetic models with enhanced osteoclastogenesis, such as very low density lipoprotein receptor (VLDLR) knockout mice vs. littermate wild-type controls, exhibited lower mTORC1 activity in their bone marrow osteoclast differentiation cultures (Fig. 1a, Supplementary Fig. 1a–b)^39^. In contrast, mouse genetic models with attenuated osteoclastogenesis, such as reelin (Reln) knockout mice vs. littermate wild-type controls, exhibited higher mTORC1 activity in their osteoclast differentiation cultures (Fig. 1b–d, Supplementary Fig. 1c-l).

To pharmacologically probe the roles of mTORC1 in osteoclastogenesis, we treated osteoclast differentiation cultures with various amount of rapamycin, a potent inhibitor of mTORC1^40^. In this assay, wildtype bone marrow osteoclast precursors were first expanded with M-CSF for 3 days, and then differentiated into osteoclasts with RANKL for 3 days with rapamycin added at indicated concentration (Fig. 1e). Osteoclast differentiation was quantified by osteoclast markers such as TRAP (tartrate-resistant acid phosphatase) (Fig. 1e); osteoclast precursor proliferation was measured by BrdU incorporation (Fig. 1f). At higher dose (30 nM), rapamycin severely impaired osteoclast differentiation (Fig. 1e), correlated with drastic inhibition of proliferation (Fig. 1f) as previously reported^41^. Interestingly, at lower dose (0.05 nM), rapamycin instead enhanced osteoclast differentiation (Fig. 1e), with only modest inhibition of proliferation (Fig. 1f). To dissect whether the pro-osteoclastogenic effects of low-dose rapamycin occurred during proliferation or differentiation, we restricted rapamycin treatment to the first 3 days of M-CSF treatment or the latter 3 days of RANKL treatment, and compared with all 6-day treatment. The results showed that low dose rapamycin enhanced osteoclast differentiation when added during the differentiation stage only (days 4–6) (Fig. 1e), but less optimal or no effect when added during the proliferation stage (days 1–3) or when added throughout the process (days 1–6) (Supplementary Fig. 1m, n). Moreover, enhanced osteoclast differentiation by low dose rapamycin resulted from mTORC1 signaling inhibition (Fig. 1g, h). Together, these findings suggest that mTORC1 functions as a switch in osteoclastogenesis to promote proliferation but must be downregulated for differentiation to occur, and the effects of rapamycin are dosage-dependent.

### Early mTORC1 deletion in HSCs impairs osteoclast precursors

**Fig. 2:**
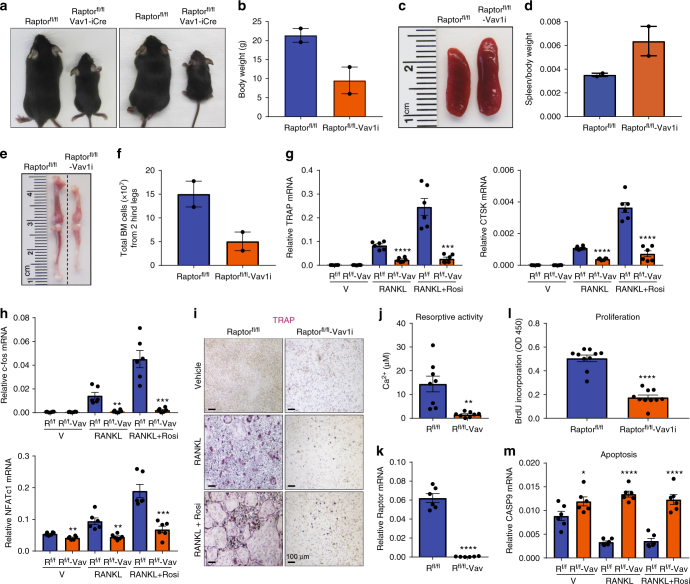
Early mTORC1 deletion in HSCs impairs osteoclast precursor proliferation and survival. Analyses of Raptor^fl/fl^-Vav1-iCre cKO mice and littermate controls. **a** Images of the pups at 6 weeks of age (*n* = 2). **b** Pup body weight at 6 weeks of age (*n* = 2). **c** Representative image of spleen at 6 weeks of age. **d** Quantification of spleen/body weight ratio at 6 weeks of age (*n* = 2). **e** Representative image of bone length at 6 weeks of age. **f** Total bone marrow cells in 6 weeks old mice (*n* = 2). **g**, **h** Expression of osteoclast markers (**g**) and osteoclastogenic transcription factors (**h**) in bone marrow osteoclast differentiation cultures (*n* = 6). Rosi rosiglitazone. **i** Representative images of TRAP-stained bone marrow osteoclast differentiation cultures. **j** Osteoclast resorptive activity measured by calcium release from bone plates (*n* = 8). **k** Raptor expression in bone marrow osteoclast differentiation cultures (*n* = 6). **l** Osteoclast precursor proliferation by BrdU incorporation (*n* = 10). **m** Caspase 9 expression in bone marrow osteoclast differentiation cultures (*n* = 6). Error bars, SEM; *p < 0.05; **p < 0.01; ***p < 0.005; ****p < 0.001; n.s. non-significant. R raptor

To genetically examine the biphasic functions of mTORC1 during osteoclastogenesis in vivo, we generated conditional knockout (cKO) mice for the essential mTORC1 regulatory subunit Raptor^35,42^. We first deleted Raptor in hematopoietic stem cells (HSCs) by breeding Raptor^fl/fl^ mice with Vav1 codon-improved Cre (iCre) mice, termed Vav1-iCre^43^. Raptor deletion by Vav1-iCre resulted in early lethality from embryonic stage to postnatal day 2, consistent with a previous report^44^. Through many breeding pairs, we could only obtain two Raptor^fl/fl^;Vav1-iCre cKO pups, with an estimated survival rate to adulthood of <1%. The two Raptor^fl/fl^-Vav1-iCre cKO mice exhibited growth retardation, shorter femurs and tibiae, splenomegaly (Fig. 2a–e), and reduced bone marrow cellularity (Fig. 2f, Supplementary Table 1). We next tested whether these mice had any defect in osteoclastogenesis ex vivo. Osteoclast differentiation from the bone marrow of Raptor^fl/fl^-Vav1-iCre cKO mice was completely blunted, shown by osteoclast markers (Fig. 2g), osteoclastogenic transcription factors (Fig. 2h), TRAP staining (Fig. 2i), and resorptive activity (Fig. 2j). Vav1-iCre conferred 99% of Raptor deletion in osteoclast differentiation cultures (Fig. 2k). This failure of osteoclastogenesis correlated with a 65% reduction in precursor proliferation (Fig. 2l), along with elevated osteoclast apoptosis (Fig. 2m).

### Late mTORC1 deletion enhances osteoclast differentiation

**Fig. 3:**
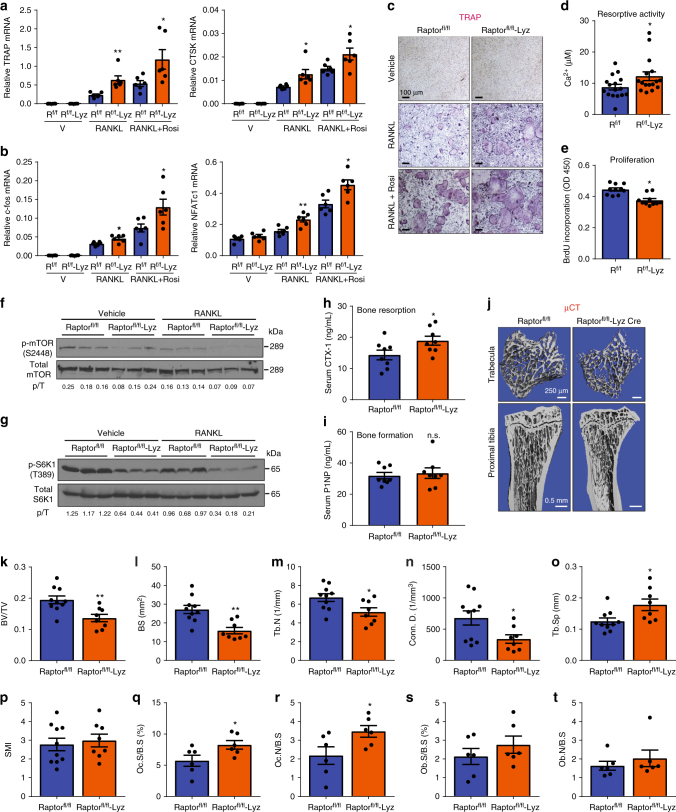
Late mTORC1 deletion in myeloid lineage enhances osteoclastogenesis. Analyses of Raptor^fl/fl^-Lyz-Cre cKO mice and littermate controls. **a**, **b** Expression of osteoclast markers (**a**) and osteoclastogenic transcription factors (**b**) in bone marrow osteoclast differentiation cultures (*n* = 6). **c** Representative images of TRAP-stained osteoclast differentiation cultures. **d** Osteoclast resorptive activity measured by calcium release from bone plates (*n* = 16). **e** Osteoclast precursor proliferation by BrdU incorporation (*n* = 10). **f**, **g** mTORC1 signaling in bone marrow osteoclast differentiation cultures from Raptor^fl/fl^-Lyz mice or littermate controls 50 h after RANKL treatment. **f** Phosphorylation of mTOR; **g** Phosphorylation of S6K1. p/T, ratio of phosphorylated/total protein. **h**–**t** Bone phenotype in 3-month-old male mice. **h** Serum CTX-1 bone resorption marker (*n* = 8). **i** Serum P1NP bone formation marker (*n* = 8). **j** Representative μCT images of the trabecular bone of the tibial metaphysis (top) and the entire proximal tibia (bottom). **k**–**p** Quantification of trabecular bone volume and architecture in proximal tibiae by μCT (*n* = 8–10). **k** BV/TV bone volume/tissue volume ratio; **l** BS bone surface; **m** Tb.N trabecular number; **n** Conn. D. connectivity density; **o** Tb.Sp trabecular separation; **p** SMI structure model index. **q**–**t** Bone histomorphometry of distal femurs (*n* = 6). Oc.S osteoclast surface, B.S bone surface, Oc.N osteoclast number, Ob.S osteoblast surface, Ob.N osteoblast number. Error bars, SEM; *, *p* < 0.05; **, *p* < 0.01; ***, *p* < 0.005; ****, *p* < 0.001; n.s. non-significant. R raptor. Full-size scans of immunoblots are shown in Supplementary Fig. 7

To specifically eliminate Raptor in the myeloid lineage, we next deleted Raptor using lysozyme (Lyz) Cre. Raptor^fl/fl^-Lyz cKO mice did not exhibit any gross phenotype with normal body weight, bone marrow cellularity, and spleen weight compared with Raptor^fl/fl^ littermate controls (Supplementary Fig. 2a–c, Supplementary Table 2). In contrast to Raptor^fl/fl^-Vav1-iCre, we observed increased osteoclast differentiation in Raptor^fl/fl^-Lyz cKO bone marrow cultures, shown by osteoclast markers (Fig. 3a), osteoclastogenic transcription factors (Fig. 3b), TRAP staining (Fig. 3c), and resorptive activity (Fig. 3d). Lyz-Cre conferred 63% Raptor deletion in the osteoclast differentiation culture (Supplementary Fig. 2d). The enhanced osteoclastogenesis in Raptor^fl/fl^-Lyz cultures correlated with 16% decreased osteoclast precursor proliferation (Fig. 3e) and attenuated osteoclast apoptosis (Supplementary Fig. 2e). As expected, mTORC1 signaling was reduced in Raptor^fl/fl^-Lyz osteoclast cultures (Fig. 3f, g). In contrast, NFκB activity at the same time point was unaltered, measured by IκBα level (Supplementary Fig. 2f, g). Consistent with our ex vivo osteoclastogenesis assay, ELISA analyses showed that the serum bone resorption marker CTX-1 (carboxy-terminal telopeptides of type I collagen) was higher in Raptor^fl/fl^-Lyz cKO mice (Fig. 3h). Serum bone formation marker P1NP (amino-terminal propeptide of type I procollagen) was unaltered (Fig. 3i). µCT of the trabecular bones in proximal tibiae revealed that Raptor^fl/fl^-Lyz cKO mice displayed a low-bone-mass phenotype (Fig. 3j–p), with decreased bone volume/tissue volume ratio (BV/TV) (Fig. 3k), bone surface (BS) (Fig. 3l), trabecular number (Tb.N) (Fig. 3m), and connectivity (Conn.) (Fig. 3n), as well as increased trabecular separation (Tb.Sp) (Fig. 3o) and structure model index (SMI) (Fig. 3p). In accordance with serum bone markers, static bone histomorphometry showed that femurs of Raptor^fl/fl^-Lyz cKO mice had higher osteoclast number and surface (Fig. 3q, r); osteoblast number and surface also trended higher although not significant (Fig. 3s, t). Dynamic histomorphometry by double calcein labeling showed increased bone formation rate and mineral apposition rate (Supplementary Fig. 2h–j), consistent with the results that osteoblast differentiation was enhanced (Supplementary Fig. 2k, l). Nonetheless, the low-bone-mass phenotype in Raptor^fl/fl^-Lyz cKO mice (Fig. 3j–p) indicated that the increased bone resorption was dominant over increased bone formation. Collectively, the results from these two genetic models support the biphasic roles of mTORC1 to promote precursor proliferation but impede osteoclast differentiation; mTORC1 signaling and cell proliferation need to be downregulated for efficient osteoclast differentiation.

### Excessive mTORC1 inhibits osteoclast differentiation

**Fig. 4:**
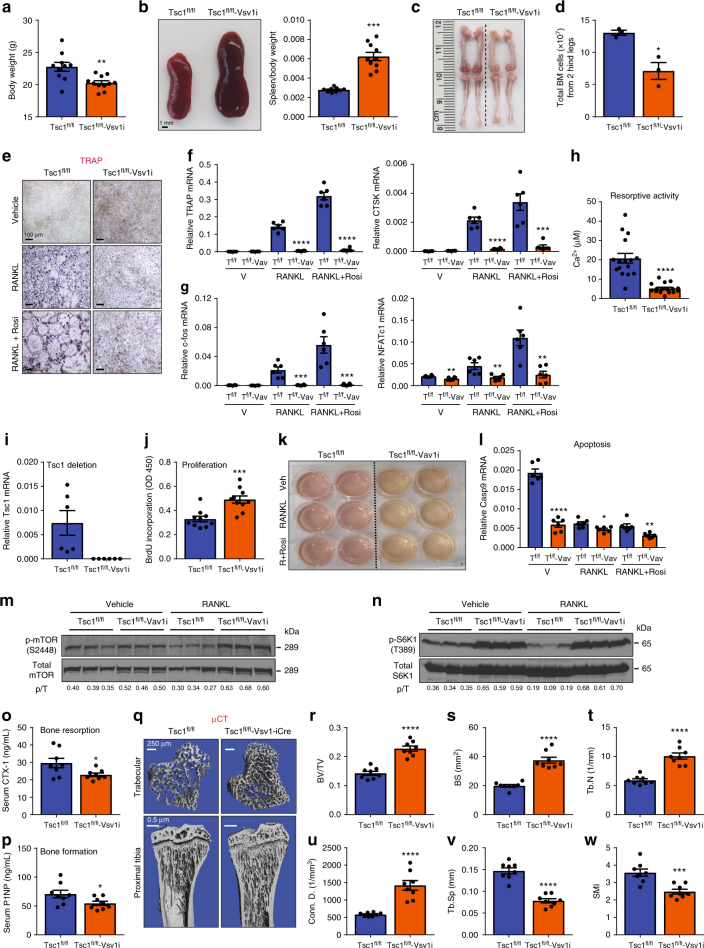
Constitutive mTORC1 activation in HSCs blunts osteoclastogenesis. Analyses of Tsc1^fl/fl^-Vav1-iCre cKO mice and littermate controls. **a** Pup body weight at 2 months old (*n* = 10–11). **b** Representative image of spleen at 2 months old (left). Quantification of spleen/body weight ratio (*n* = 10–11) (right). **c** Representative image of bone length in 2 months old pups. **d** Total bone marrow cells in 2 months old pups (*n* = 3). **e** Representative images of TRAP-stained osteoclast differentiation cultures. **f**, **g** Expression of osteoclast markers (**f**) and osteoclastogenic transcription factors (**g**) in bone marrow osteoclast differentiation cultures (*n* = 6). **h** Osteoclast resorptive activity measured by calcium release from bone plates (*n* = 16). **i** Tsc1 expression in bone marrow osteoclast differentiation cultures (*n* = 6). **j** Osteoclast precursor proliferation by BrdU incorporation (*n* = 10). **k** Representative image of culture media color changes in Tsc1^fl/fl^-Vav1-iCre cKO bone marrow cultures. **l** Caspase 9 expression in bone marrow osteoclast differentiation cultures (*n* = 6). **m**, **n** mTORC1 signaling in bone marrow osteoclast differentiation cultures 60 h after RANKL treatment. **m** Phosphorylation of mTOR; **n** Phosphorylation of S6K1. p/T ratio of phosphorylated/total protein. **o** Serum CTX-1 bone resorption marker (*n* = 8). **p** Serum P1NP bone formation marker (*n* = 8). **q** Representative μCT images of the trabecular bone of the tibial metaphysis (top) and the entire proximal tibia (bottom). **r**–**w** Quantification of trabecular bone volume and architecture in proximal tibiae by μCT (*n* = 8). **r** BV/TV bone volume/tissue volume ratio, **s** BS bone surface, **t** Tb.N trabecular number, **u** Conn. D. connectivity density, **v** Tb.Sp trabecular separation, **w** SMI structure model index. Error bars, SEM; *p < 0.05; **p < 0.01; ***p < 0.005; ****p < 0.001; n.s. non-significant. T Tsc1. Full-size scans of immunoblots are shown in Supplementary Fig. 8

As a complementary strategy to the loss-of-function models, we also generated gain-of-function models by conditionally activating mTORC1 via the deletion of Tsc1 suppressor. Tsc1 deletion in hematopoietic stem cells in Tsc1^fl/fl^-Vav1-iCre cKO mice led to decreased body weight (Fig. 4a), splenomegaly (Fig. 4b), shorter femurs and tibiae (Fig. 4c), as well as defects in bone marrow cellularity (Fig. 4d, Supplementary Table 3). Bone marrow osteoclast differentiation was severely blunted for Tsc1^fl/fl^-Vav1-iCre cKO cultures, shown by TRAP staining (Fig. 4e), osteoclast markers (Fig. 4f), osteoclastogenic transcription factors (Fig. 4g), and resorptive activity (Fig. 4h). Tsc1 deletion efficiency by Vav1-iCre was >99% (Fig. 4i). The impaired osteoclastogenesis in Tsc1^fl/fl^-Vav1-iCre cultures correlated with increased precursor proliferation (Fig. 4j, k) and decreased osteoclast apoptosis (Fig. 4l), as the result of constitutive mTORC1 signaling (Fig. 4m, n). Consistent with our ex vivo osteoclastogenesis assay, ELISA showed that serum CTX-1 was decreased in Tsc1^fl/fl^-Vav1-iCre cKO mice (Fig. 4o). Serum P1NP was also lower possibly due to a compensatory effect due to the coupling mechanisms (Fig. 4p). Nonetheless, the reduction in bone resorption was dominant as µCT revealed a high-bone-mass phenotype in the Tsc1^fl/fl^-Vav1-iCre cKO mice (Fig. 4q–w).

**Fig. 5:**
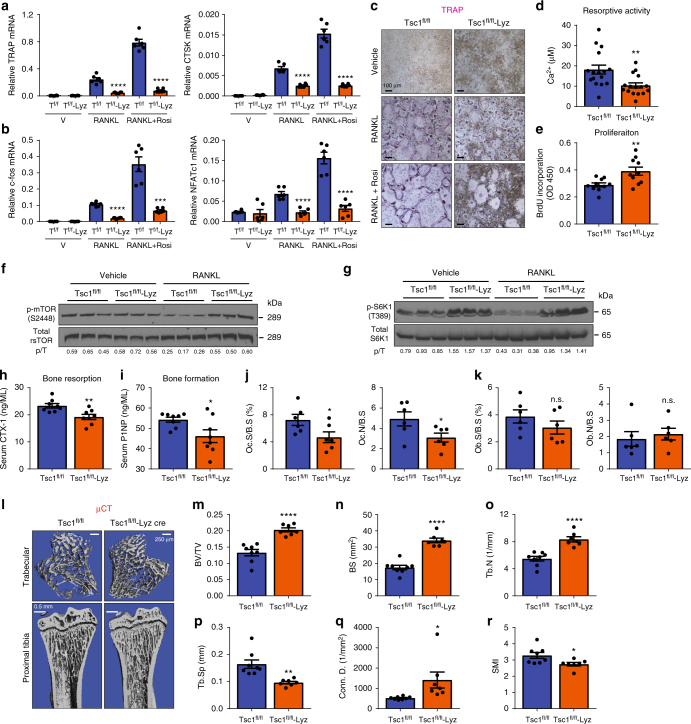
Constitutive mTORC1 activation in myeloid lineage impedes osteoclast differentiation. Analyses of Tsc1^fl/fl^-Lyz-Cre cKO mice and littermate controls. **a**, **b** Expression of osteoclast markers (**a**) and osteoclastogenic transcription factors (**b**) in bone marrow osteoclast differentiation cultures (*n* = 6). **c** Representative images of TRAP-stained osteoclast differentiation cultures. **d** Osteoclast resorptive activity measured by calcium release from bone plates (*n* = 16). **e** Osteoclast precursor proliferation by BrdU incorporation (*n* = 10). **f**, **g** mTORC1 signaling in bone marrow osteoclast differentiation cultures 60 h after RANKL treatment. **f** Phosphorylation of mTOR; **g** Phosphorylation of S6K1. p/T ratio of phosphorylated/total protein. **h**–**r** Bone phenotype in 2-month-old male mice. **h** Serum CTX-1 bone resorption marker (*n* = 8). **i** Serum P1NP bone formation marker (*n* = 8). **j** Bone histomorphometry of distal femurs (*n* = 6). Oc.S osteoclast surface, B.S bone surface, Oc.N osteoclast number. **k** Bone histomorphometry of distal femurs (*n* = 6). Ob.S osteoblast surface, B.S bone surface, Ob.N osteoblast number. **l** Representative μCT images of the trabecular bone of the tibial metaphysis (top) and the entire proximal tibia (bottom). **m**–**r** Quantification of trabecular bone volume and architecture in proximal tibiae by μCT (*n* = 8). **m** BV/TV bone volume/tissue volume ratio, **n** BS bone surface, **o** Tb.N trabecular number, **p** Tb.Sp trabecular separation, **q** Conn. D. connectivity density, **r** SMI structure model index. Error bars, SEM; *p < 0.05; **p < 0.01; ***p < 0.005; ****p < 0.001; n.s. non-significant. T Tsc1. Full-size scans of immunoblots are shown in Supplementary Fig. 9

To more specifically determine the effects of mTORC1 activation in the myeloid lineage, we conditionally deleted Tsc1 with Lyz-Cre. Tsc1^fl/fl^-Lyz cKO mice had normal body weight and femur/tibia lengths (Supplementary Fig. 3a, b), but also exhibited splenomegaly (Supplementary Fig. 3c, d) and mild defects in bone marrow cellularity (Supplementary Fig. 3e, Supplementary Table 4). Osteoclastogenesis from Tsc1^fl/fl^-Lyz cKO bone marrow was also impaired, shown by osteoclast markers (Fig. 5a), osteoclastogenic transcription factors (Fig. 5b), TRAP staining (Fig. 5c), and resorptive activity (Fig. 5d). Lyz-Cre conferred 92% Tsc1 deletion in the osteoclast differentiation cultures (Supplementary Fig. 3f). Once again, the diminished osteoclastogenesis in Tsc1^fl/fl^-Lyz cultures correlated with increased osteoclast precursor proliferation (Fig. 5e, Supplementary Fig. 3g) and decreased osteoclast apoptosis (Supplementary Fig. 3h), owing to elevated mTORC1 signaling (Fig. 5f, g). NFκB activity at the same time point was unaffected (Supplementary Fig. 3i, j). ELISA showed that Tsc1^fl/fl^-Lyz cKO mice also had a decrease in CTX-1 (Fig. 5h), accompanied by a reduction in P1NP (Fig. 5i). Static bone histomorphometry showed that femurs of Tsc1^fl/fl^-Lyz cKO mice exhibited lower osteoclast number and surface (Fig. 5j); osteoblast number and surface also trended lower but not significant (Fig. 5k). Dynamic histomorphometry by double calcein showed that bone formation rate and mineral apposition rate were decreased in Tsc1^fl/fl^-Lyz cKO mice (Supplementary Fig. 3k, m), in line with the reduced osteoblast differentiation (Supplementary Fig. 3n, o). µCT revealed a high-bone-mass phenotype in the Tsc1^fl/fl^-Lyz cKO mice, suggesting that the reduced bone resorption was dominant over the reduced bone formation (Fig. 5l–r). Together, these results reveal that constitutive mTORC1 activation prevents osteoclast differentiation and mTORC1 is a suppressor of RANKL signaling.

### Calcineurin inhibits mTORC1 via dephosphorylation

**Fig. 6:**
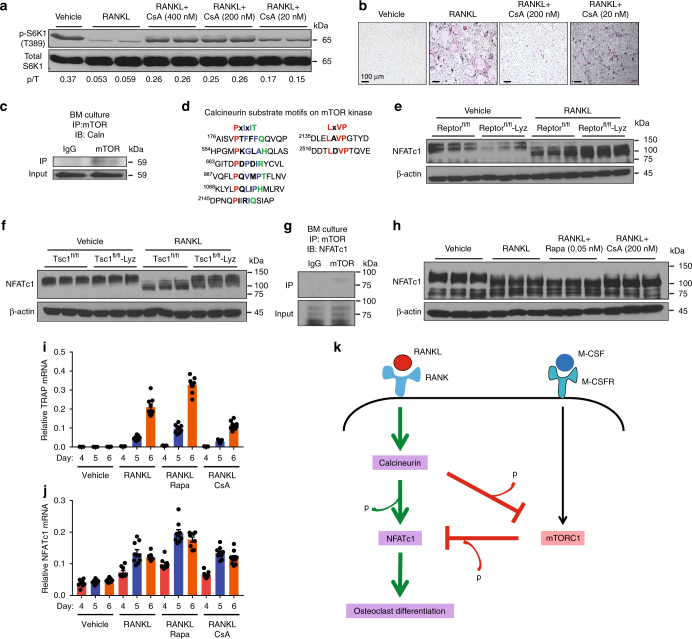
Calcineurin inhibits mTORC1 signaling to enhance NFATc1 activation. **a** mTORC1 signaling in osteoclast differentiation cultures 60 h after RANKL treatment with or without CsA, measured by S6K1 phosphorylation. p/T ratio of p-S6K1/total S6K1. **b** Representative images of TRAP-stained osteoclast differentiation cultures on day 9. **c** Western blotting of calcineurin from mTOR immunoprecipitates. **d** Bioinformatic analysis of PxIxIT and LxVP motifs in mTOR kinase. **e**, **f** NFATc1 mobility shifts in bone marrow osteoclast differentiation cultures 60 h after RANKL treatment. **e** Raptor^fl/fl^-Lyz cKO mice. **f** Tsc1^fl/fl^-Lyz cKO mice. **g** Western blot of NFATc1 from mTOR immunoprecipitates. **h** NFATc1 mobility shifts in bone marrow osteoclast differentiation cultures 48 h after RANKL treatment with or without rapamycin (Rapa) or CsA. **i**, **j** Expression of TRAP (**i**) and NFATc1 (**j**) during a time course of bone marrow osteoclast differentiation with rapamycin (Rapa) or CsA (*n* = 9). **k** A working model of how the calcineurin ⊣ mTORC1 ⊣ NFATc1 cascade regulates osteoclast differentiation. Error bars, SEM; *p < 0.05; **p < 0.01; ***p < 0.005; ****p < 0.001; n.s. non-significant. Full-size scans of immunoblots are shown in Supplementary Fig. 10

We next investigated the mechanisms for how RANKL downregulates mTORC1 signaling. Our data showed that mTOR phosphorylation was diminished upon RANKL stimulation (Figs. 3f and 5f), suggesting that some type of serine-threonine phosphatase downstream of RANKL signaling may be responsible. Because calcineurin (also known as PP2B or PP3) is a RANKL-activated serine-threonine phosphatase, we decided to examine whether calcineurin regulates mTORC1. First, we treated osteoclast cultures with increasing doses of cyclosporin A (CsA), a potent inhibitor of calcineurin^45^. At 20–400 nM, CsA increased mTORC1 signaling (Fig. 6a), consequently diminishing osteoclast differentiation, shown by the decreased TRAP expression (Supplementary Fig. 4a) as well as the reduced number and size of multinucleated osteoclasts (Fig. 6b). Next, our co-immunoprecipitation (co-IP) analysis revealed that calcineurin could physically bind to mTOR kinase (Fig. 6c), suggesting that mTOR may be a direct calcineurin substrate. It has been well known that substrates for calcineurin contain two short specific motifs, *PxIxIT* and *LxVP*, which are highly conserved features critical for their dephosphorylation by calcineurin (Supplementary Fig. 4b)^46^. *PxIxIT* is primary the docking site that specifies the binding to calcineurin, with proline at position 1, hydrophobic residues at position 3 and 5, and a hydrophilic residue at position 6; whereas *LxVP* is a secondary motif that directs the binding to active calcineurin. Our bioinformatics analysis identified multiple cases of both motifs in mTOR kinase (Fig. 6d), further supporting mTOR as a direct substrate for calcineurin-mediated dephosphorylation upon RANKL stimulation.

### mTORC1 inhibits NFATc1 via phosphorylation

We next examined the mechanisms for how mTORC1 kinase impedes osteoclast differentiation. NFATc1—a master transcriptional factor required for osteoclast differentiation—is activated by a reduction of phosphorylation and subsequently nuclear translocation^12,47–51^. Consistent with the literature, we could detect by western blot multiple NFATc1 bands in osteoclast precursors and a downward shift of NFATc1 bands upon RANKL stimulation, possibly due to a combination of decreased phosphorylation and increased dephosphorylation at multiple sites (Supplementary Fig. 4c). This reduced NFATc1 phosphorylation correlated with the mRNA induction of osteoclast markers such as TRAP (Supplementary Fig. 4d) and osteoclastogenic transcription factors such as NFATc1 itself (Supplementary Fig. 4e). Although calcineurin represents a major NFATc1 phosphatase^49–51^, the kinase(s) responsible for NFATc1 phosphorylation in osteoclast precursors is still unclear. Given mTORC1 is an anti-osteoclastogenic serine–threonine kinase (Figs. 1–5**)**, we hypothesize that mTORC1 phosphorylates NFATc1 during precursor proliferation stage, and the downregulation of mTORC1 activity upon RANKL stimulation permits efficient reduction of NFATc1 phosphorylation and consequently osteoclast differentiation. To test this hypothesis, we examined the effects of mTORC1 inhibition or activation on NFATc1 mobility shift in bone marrow osteoclast differentiation cultures. As the result of lower mTORC1 signaling (Fig. 3f, g), NFATc1 was shifted down in Raptor^fl/fl^-Lyz cKO osteoclast cultures vs. control cultures (Fig. 6e), indicating a reduced NFATc1 phosphorylation. In agreement, as the result of constitutive mTORC1 activation (Fig. 5f, g), NFATc1 was shifted up in Tsc1^fl/fl^-Lyz or Tsc1^fl/fl^-Vav1-iCre cKO osteoclast cultures (Fig. 6f, Supplementary Fig. 4f), indicating an elevated NFATc1 phosphorylation. Furthermore, our co-IP analyses showed that mTOR kinase could physically bind to NFATc1 in both bone marrow osteoclast precursors (Fig. 6g) and RAW264.7 osteoclast precursors (Supplementary Fig. 4g), supporting the notion that NFATc1 may be an mTORC1 substrate.

Complementary to these genetic approaches, our pharmacological experiments showed that NFATc1 in osteoclast differentiation cultures was further shifted down upon mTORC1 suppression by rapamycin, which was reverted up upon mTORC1 activation by CsA calcineurin inhibition (Fig. 6h). TRAP mRNA induction (Fig. 6i) correlated with the changes in NFATc1 mobility (Fig. 6h) rather than NFATc1 mRNA (Fig. 6j), indicating that NFATc1 regulation at protein level is more important. As a result, NFATc1 nuclear localization was enhanced by rapamycin but attenuated by CsA (Supplementary Fig. 4h, i), further supporting that mTORC1 negatively regulates NFATc1 functions possibly by increasing NFATc1 phosphorylation.

We next performed bioinformatic analyses to identify evolutionarily conserved mTORC1 phosphorylation sites in NFATc1. A previous phosphoproteomic study combining positional scanning peptide libraries and quantitative mass spectrometry has determined the consensus mTOR phosphorylation motifs, which show selectivity toward peptide substrates with proline, hydrophobic residues (L, V), and aromatic residues (F, W, Y) at +1 position (downstream of serine or threonine)^52^. This pattern of specificity at the +1 position is concordant with known mTOR phosphorylated sites (Supplementary Fig. 5a). Moreover, this mTOR phosphoproteomic study has detected an NFATc1 phosphopeptide with proline at +1 position (Supplementary Fig. 5b)^52^. According to these motifs, we identified approximately 60 potential mTORC1 phosphorylation sites in human and mouse NFATc1 (Supplementary Fig. 5c–h). To pinpoint the evolutionally conserved sites, we performed amino-acid sequence alignments of NFATc1 proteins from human and mouse, as well as four other vertebrates including rat, pig, monkey, and chimpanzee (Supplementary Fig. 5i–l). This exercise revealed 30 fully conserved sites, with 18 carrying serine-proline motifs, including the site identified in the mTOR phosphoproteomic study^52^. These results indicate that NFATc1 may be predominantly phosphorylated by mTORC1 at serine residues followed by proline (Supplementary Fig. 5i–l). Collectively, our data provide evidence for a working model in which RANKL activated calcineurin suppresses mTORC1 signaling by dephosphorylation, which in turn promotes NFATc1 functions by reducing NFATc1 phosphorylation (Fig. 6k). This new calcineurin ⊣ mTORC1 ⊣ NFATc1 signaling cascade may act in synergy with presently known mechanisms to effectively enhance osteoclast differentiation (Fig. 6k).

### Rapamycin increases bone resorption in vivo

**Fig. 7:**
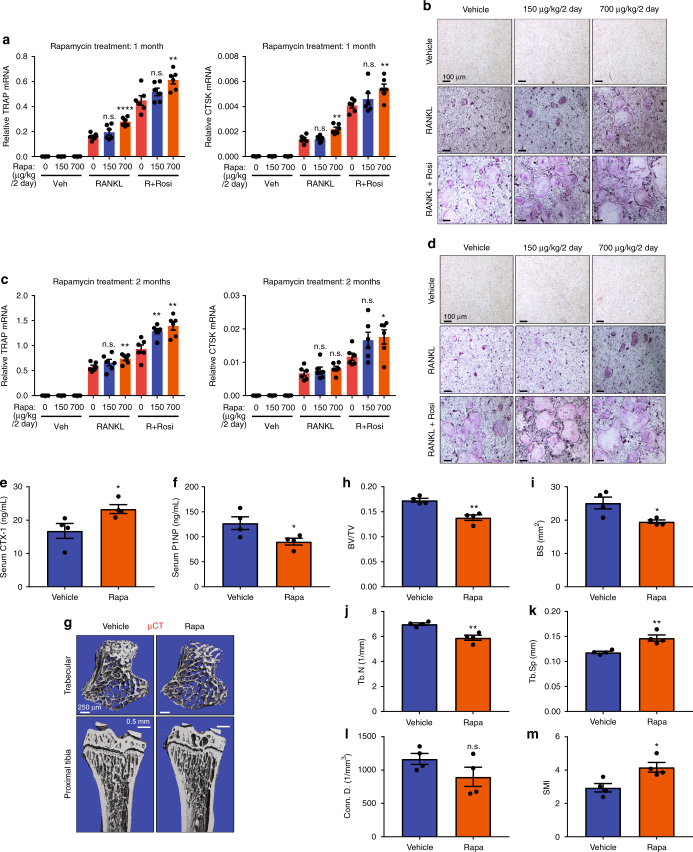
Rapamycin enhances osteoclastogenesis and bone resorption in vivo. **a**–**d** Ex vivo bone marrow osteoclast differentiation from 3 mice/group after 1 month (**a**, **b**) or 2 months (**c**, **d**) of rapamycin or vehicle treatment. **a**, **c** Expression of osteoclast markers in differentiation cultures (*n* = 6). Representative results from 3 mice/group. (**b**, **d**) Representative images of TRAP-stained differentiation cultures. Rosi, rosiglitazone. **e**–**m** In vivo bone phenotype in mice treated with vehicle control or rapamycin (Rapa) at 700 μg/kg every 2 days. **e** Serum CTX-1 bone resorption marker (*n* = 4). **f** Serum P1NP bone formation marker (*n* = 4). **g** Representative μCT images of the trabecular bone of the tibial metaphysis (top) and the entire proximal tibia (bottom). **h**–**m** Quantification of trabecular bone volume and architecture in proximal tibiae by μCT (*n* = 4). **h** BV/TV bone volume/tissue volume ratio, **i** BS bone surface, **j** Tb.N trabecular number, **k** Tb.Sp trabecular separation, **l** Conn. D. connectivity density, **m** SMI structure model index. Error bars, SEM; *p < 0.05; **p < 0.01; ***p < 0.005; ****p < 0.001; n.s. non-significant

Our ex vivo findings show that rapamycin exerts dosage-dependent effects on osteoclastogenesis, with inhibition by high dose but augmentation by low dose (Fig. 1e–g). In light of the clinical usage of rapamycin as immunosuppressant and cancer drugs, such as the FDA-approved Sirolimus and Everolimus, we performed in vivo rapamycin treatment to examine the effects on osteoclastogenesis, bone resorption and bone mass. The recommended dose for Sirolimus and Everolimus is ~6–20 mg/day, which translates to ~60–400 μg/kg/day for an individual with 50–100 kg body weight. Thus, we treated wildtype mice with rapamycin at similar doses − 75 μg/kg/day (injecting 150 μg/kg every 2 days) and 350 μg/kg/day (injecting 700 μg/kg every 2 days). At both doses, in vivo rapamycin treatment enhanced bone marrow osteoclast differentiation after both 1 month and 2 months (Fig. 7a–d). Consistent with these observations in ex vivo bone marrow osteoclastogenesis assays, in vivo analyses showed that rapamycin treatment increased bone resorption (Fig. 7e) and decreased bone formation (Fig. 7f), leading to a lower bone mass (Fig. 7g–m). These results reveal potential bone loss effects of rapamycin treatment at clinically relevant dosage.

## Discussion

Our study using a series of gain- and loss-of-function mouse genetic models uncovers mTORC1 as a key proliferation-to-differentiation switch during osteoclastogenesis. mTORC1 is not only required but also must be dynamically regulated so that it is activated during proliferation but inactivated during differentiation. Our examination of the pharmacological effects of rapamycin in vitro and in vivo reveals a dosage-dependent regulation of osteoclastogenesis by rapamycin. Importantly, our mechanistic investigation identifies a previously unrecognized but fundamentally important calcineurin ⊣ mTORC1 ⊣ NFATc1 phosphorylation-regulatory signaling cascade that will broadly impact many fields such as bone, immunity, and cancer.

Our data suggest that mTORC1 is a direct calcineurin substrate, because our co-IP showed that calcineurin can bind to mTOR kinase, and mTOR contains several *PxIxIT* and *LxVP* motifs that are required for functional calcineurin substrates (Fig. 6c, d, Supplementary Fig. 4b). In contrast, our co-IP could not detect calcineurin binding to S6K1 kinase, and our bioinformatic analysis did not find any *PxIxIT* or *LxVP* motif on S6K1, suggesting that mTOR rather than S6K1 is a direct calcineurin substrate. Nonetheless, alternative or complementary mechanisms may exist, for example, calcineurin may use mTOR kinase as an anchor to dephosphorylate other mTORC1 subunits.

Similarly, our data suggest that NFATc1 is a direct mTORC1 substrate, because our genetic and pharmacological studies support a functional role of mTOR kinase in promoting NFATc1 phosphorylation (Fig. 6e, f, h, Supplementary Fig. 4f); our co-IP and bioinformatic analyses support the physical binding of mTOR kinase to NFATc1 as well as their enzyme substrate relationship (Fig. 6g, Supplementary Fig. 5). Nonetheless, mTORC1 may also phosphorylate other substrates to confer alternative or complementary mechanisms for its regulation of both MCSF-mediated osteoclast precursor proliferation and RANKL-mediated osteoclast differentiation. We have observed decreased NFATc1 phosphorylation upon Raptor deletion before RANKL treatment (Fig. 6e). NFATc1 has been shown to regulate the proliferation of both hematopoietic and non-hematopoietic cell types^53,54^, thus it is likely that mTORC1 phosphorylation of NFATc1 also impact osteoclast progenitor proliferation. In addition, many mTORC1 downstream targets have been shown to contribute to its regulation of cell growth and proliferation; hence it is possible that these other pathways are also involved. Furthermore, our data suggest that mTOR is only one of the kinases that phosphorylate NFATc1. As shown in Fig. 6e, NFATc1 was still partially phosphorylated in the absence of Raptor, indicating that other kinases were also involved. Raptor deletion shifted the balance by decreasing phosphorylated NFATc1 before RANKL treatment and increasing dephosphorylated NFATc1 after RANKL treatment. Besides NFATc1, other signaling pathways induced by RANKL, such as AP-1 and NFkB, are also required for a robust osteoclastogenesis. Therefore, partial NFATc1 dephosphorylation due to Raptor deletion was not able to lead to pronounced RANKL-independent osteoclastogenesis (Fig. 3a–c).

Upon binding of RANKL to its receptor RANK, the resulted complex triggers activation of various immediate downstream signaling cascades such as mitogen-activated protein kinases (MAPK) including p38, c-Jun N-terminal kinase (JNK) and extracellular signal-regulated kinase (ERK), as well as transcription factors such as NFκB and AP-1^47^. These pathways in the triggering phase are activated within an hour of RANKL stimulation. The transition of triggering phase to amplifying phase requires the induction of NFATc1 activity, which allows further downstream signaling. During the amplifying phase starting around 24 h after RANKL stimulation, intracellular Ca^2+^ levels oscillate, and activate the Ca^2+/^calmodulin-dependent phosphatase calcineurin, which dephosphorylates NFATc1 and induces NFATc1 nuclear translocation, leading to the increased transcription of NFATc1 target genes^14,15^. Consistent with this notion, we did not observe any significant changes of NFATc1 shift or S6K1 phosphorylation level at 24 h of RANKL stimulation (Supplememtary Fig. 1o, p). However, we began to see the downward shift of NFATc1 and the decreased phosphorylation of S6K1 at ~36 h, and the best effects for both were observed between 48 and 60 h after RANKL treatment. As osteoclasts became more mature at 72 h of RANKL stimulation, we could not detect p-S6K1 (Supplementary Fig. 1q). Therefore, we chose 48–60 h window for our experiments to see clear effects on mTORC1 and NFATc1. At this time window, we did not observe any change in IκBα levels in the mutant mice (Supplementary Fig. 2f, g, 3i, j). However, it is possible that other signaling pathways such as NFκB may be altered as direct or indirect consequences at other time points.

Our pharmacological studies in vivo using clinically relevant dose of rapamycin revealed a pro-osteoclastogenic and resorption-enhancing effect, contributing to a decreased bone mass. Nonetheless, in addition to the osteoclast-autonomous signaling supported by our study, the bone loss effects of rapamycin may be also contributed by other tissues and cell types, such as the reported increased RANKL but decreased OPG expression from marrow stromal cells^55^. Moreover, it has been shown that the effects of rapamycin and mTOR on osteoclastogenesis may be influenced by the bioenergetics microenvironment^56^.

Consistent with our current findings using in vivo mouse models (Figs. 1–5**)**, a previous in vitro study has also shown that mTORC1 inhibition by rapamycin enhances osteoclast differentiation from RAW264.7 macrophages^57^. In accordance with our current findings of increased osteoclastogenesis in rapamycin-treated mice (Fig. 7), a previous pharmacological study has also shown that rapamycin led to increased bone remodeling and bone loss in healthy male rats^58^. Clinically, it has been observed that in osteoporotic women with elevated osteoclast activity and bone resorption, mTOR expression was lower by three-fold in their peripheral blood cells, which include circulating osteoclast precursors^59^. Sirolimus has been shown to increase bone resorptive marker in renal transplant patients, leading to high bone turnover osteopenia^60^. Moreover, bone pain and bone necrosis have been widely reported as a common side effect of sirolimus. Nonetheless, several studies have also shown that sirolimus reduces osteoclastic bone resorption^61–64^. Despite these clinical observations, there has not been a deep molecular and cellular understanding for how rapamycin affects osteoclastogenesis and bone turnover. Highly clinically relevant, our study is the first providing mechanistic basis for these seemingly contradictory reports via revealing the biphasic regulation by mTORC1 and the dosage-dependent effects of rapamycin on osteoclastogenesis, genetically and pharmacologically, in vitro and in vivo. The long-term effects of rapamycin drugs on bone are still unclear due to the limited clinical information to date^65^. Our work will facilitate future clinical studies to carefully examine the dosage-dependent impact of rapamycin on skeletal fitness, and select the optimal dose range to prevent any potential bone loss deleterious effects from rapamycin treatment.

Despite the biological and clinical importance, there are only a few reports on mTORC1 and osteoclastogenesis, including two recent studies presenting conflicting results^36,37^. However, these studies could not provide a unifying explanation for the contradictory clinical observations. In one study, Dai et al. deleted Raptor with Cathepsin K-Cre (Ctsk-Cre) to conclude that inactivation of mTORC1 signaling in osteoclast increases bone mass by inhibiting osteoclast differentiation in mice. This observation is in line with earlier work showing that mTORC1 promotes osteoclast precursor proliferation upon stimulation by M-CSF^36,41,61,66^. However, Ctsk-Cre is known to be non-specific, and often causes germline flox recombination and gene deletion^67^. This raises significant concerns regarding their reported in vivo bone phenotypes. Moreover, Dai et al. treated bone marrow cells with rapamycin at 1–100 nM concentration to conclude that rapamycin inhibits osteoclast differentiation in vitro. However, IC_50_ for rapamycin is ~0.1 nM^68–70^. The 1–100 nM concentration they used is considered high, and may not be clinically relevant.

In another study, Zhang et al. deleted Raptor or Tsc1 with LyzM-Cre to inhibit or activate mTORC1 signaling, respectively^37^. In agreement with this study, we observed similar in vivo bone phenotypes as well as ex vivo osteoclast differentiation using LyzM-Cre. However, Zhang et al. did not examine the pharmacological effects of rapamycin in vivo to address the clinically observed high bone turnover and bone loss; they only treated the in vitro osteoclast culture from Tsc1 cKO mice with rapamycin to show rescuing effects. In addition, using only LyzM-Cre to eliminate Raptor in myeloid lineage failed to reveal the requirement of mTORC1 signaling in osteoclastogenesis by promoting precursor proliferation and survival, and contradicted the inhibition of osteoclast differentiation by high dose of rapamycin treatment. Thus, we used additional Vav1-iCre to delete Raptor or Tsc1 in hematopoietic stem cells to reveal the consequences of early genetic deletion and the relevance of complete mTOR inhibition by high-dose rapamycin. Pharmacologically and genetically, our data demonstrate that mTORC1 acts as a dual regulator to control the proliferation-to-differentiation switch during osteoclastogenesis, which is required for precursor proliferation but must be downregulated for differentiation.

Zhang et al. concluded that mTORC1 inhibits NF-kB/NFATc1 signaling to prevent osteoclast differentiation, but did not provide a clear mechanism for how mTORC1 inhibits NFATc1^37^. First, it is well known that NFATc1 is activated by calcineurin-mediated dephosphorylation and subsequent nuclear translocation^8,9,13–15^. Consistent with this notion, our data demonstrated that NFATc1 protein mobility is shifted downward upon activation by RANKL and dephosphorylation by calcineurin, and shifted further downward with low dose of rapamycin. This pharmacological observation by rapamycin treatment could be genetically replicated ex vivo with our Raptor^fl/fl^-Lyz cKO mice. Conversely, we also demonstrated the reverse for an upward shift of NFATc1 by cyclosporin A (CsA), which was also genetically replicated with Tsc1^fl/fl^-Lyz and Tsc1^fl/fl^-Vav-iCre cKO mice. However, Zhang et al. did not examine the dephosphorylation and phosphorylation shift of NFATc1 either in their LyzM-Cre cKO mice or with rapamycin. Moreover, Zhang et al. showed a single band of NFATc1 protein with higher intensity in Raptor-LyzM and lower intensity in Tsc1-LyzM at the same position of NFATc1 protein from wildtype mice—these data are inconsistent with the fact that NFATc1 is dephosphorylated by calcineurin (early established findings) or phosphorylated by mTORC1 (our new findings). In addition to revealing the functional significance of mTORC1 in phosphorylating and inhibiting NFATc1, we have also provided compelling evidence for a direct kinase–substrate relationship between mTOR and NFATc1 by demonstrating their physical interaction and identifying multiple evolutionarily conserved mTOR phosphorylation motifs in NFATc1. We found no significant change in NFκB signaling. Thus, our study is the first to our knowledge to reveal the novel molecular mechanism of NFATc1 inhibition by mTORC1-mediated phosphorylation, which will exert broad impact on multiple fields including bone, immunology, and cancer.

Furthermore, our findings suggest that calcineurin is a phosphatase that inactivates mTORC1 upon RANKL stimulation by demonstrating a physical interaction between calcineurin and mTOR, as well as identifying calcineurin dephosphorylation motifs in mTOR. Pharmacologically, we showed that calcineurin inhibition by CsA enhances mTORC1 signaling. To our knowledge, this finding is the first to show such key regulation, which will be important in not only osteoclast but also numerous other cell types regulated by calcium and mTORC1 signaling. Together, our study uncovered a novel phosphorylation/dephosphorylation signaling cascade during osteoclastogenesis that is comprised of calcineurin ⊣ mTORC1 ⊣ NFATc1, providing important insights to both mechanisms (biphasic functions) and pharmacological treatment (dose-dependent effects). Importantly, we treated wild-type mice with clinically relevant dose of rapamycin and observed resorption-enhancing and bone loss consequences, thus providing important understanding of the clinically observed bone damaging effects in patients.

In summary, the significance and novelty of our current study resides in the following aspects. We uncovered the biphasic and dynamic regulation by mTORC1 in osteoclast proliferation and differentiation, by establishing multiple genetic models. We identified the dosage-dependent effects of rapamycin on osteoclastogenesis, and demonstrated that low but clinically relevant dose of rapamycin treatment in vivo enhances bone resorption and causes bone loss. Finally, we identified a fundamentally important signaling cascade by which calcineurin inhibits mTORC1, thus blocking its inhibition of NFATc1, which will have broad and long-reaching impact in multiple fields such as bone, immunology and cancer. Our studies with unprecedented comprehension and precision will help to unify the field and resolve the long-standing debate over the roles of mTORC1 and rapamycin on osteoclastogenesis, providing novel mechanistic and clinical insights.

## Methods

### Mice

VLDLR^−/−^ mice^71^, Reln^−/−^ mice^72^, Tsc1 flox mice^73^, Raptor flox mice^74^, Lysozyme-Cre^75^, and Vav1-iCre transgenic mice^43^ were from Jackson Laboratory and maintained on C57BL/6 background. Mice were fed standard rodent chow ad libitum (Harlan Laboratories). To generate VLDLR^−/−^ and wildtype littermates, VLDLR^+/−^ female were bred with VLDLR^+/^^−^ male mice. To generate Reln^−/−^ and wild-type littermates, Reln^+/−^ female were bred with Reln^+/−^ male mice. To obtain Raptor^flox/flox^;Vav1-iCre (or TSC1^flox/flox^;Vav1-iCre) cKO mice, male Raptor^flox/flox^ (or TSC1^flox/flox^) mice were bred with Raptor^flox/+^;Vav1-iCre (or TSC1^flox/+^;Vav1-iCre) female mice^76^. To obtain Raptor^flox/flox^;Lysozyme-Cre (or TSC1^flox/flox^;Lysozyme-Cre) cKO mice, female Raptor^flox/flox^ (or TSC1^flox/flox^) mice were bred with Raptor^flox/flox^;Lysozyme-Cre (or TSC1^flox/flox^;Lysozyme-Cre) male mice. All experiments were conducted using littermates. Sample size estimate was based on power analyses performed using SAS 9.3 TS X64_7PRO platform. All animal experiments were approved by the Institutional Animal Care and Use Committee of UT Southwestern Medical Center.

### Bone marrow osteoclast differentiation

Osteoclasts were differentiated from bone marrow cells as previously described^38^. Briefly, bone marrow cells were differentiated with 40 ng/mL of mouse M-CSF (R&D Systems) in α-MEM containing 10% FBS for 3 days (day 1–3), then with 40 ng/mL of mouse M-CSF and 100 ng/mL of mouse RANKL (R&D Systems) for 3–7 days (day 4–10), with or without rosiglitazone (1 µM) (Cayman Chemical). TRAP (tartrate-resistant acid phosphatase) staining of osteoclasts was performed using a leukocyte acid phosphatase staining kit (Sigma). Mature osteoclasts were identified as multinucleated (>3 nuclei) TRAP^+^ cells on day 10. Osteoclast differentiation was quantified by the RNA expression of osteoclast markers on day 6 using RT-qPCR analysis. Osteoclast precursor proliferation was quantified by bromodeoxyuridine (BrdU) incorporation (GE Healthcare) as previously described^77^. Osteoclast resorptive activity from the entire culture was measured by calcium release from bone plates as previously described^78^. Bone marrow osteoblast differentiation was performed as previously described^79^. Osteoblast precursors were expanded for 4 days in Mesenchymal Stem Cell (MSC) media using a Mouse MesenCult Proliferation Kit (StemCell Technologies) before the addition of osteoblast differentiation cocktail (StemCell Technologies).

### Bone analyses

µCT was performed to evaluate bone volume and architecture using a Scanco µCT-35 instrument (SCANCO Medical) as described^77^. Bone histomorphometry was conducted using Bioquant Image Analysis software (Bioquant). Dynamic histomorphometry was performed using femurs as described^77^. Calcein (20 mg/kg) was injected into 6-week-old mice 2 and 7 days before bone collection. As a bone resorption marker, serum CTX-1 was measured with the RatLaps^TM^ EIA kit (Immunodiagnostic Systems)^80^. As a bone formation marker, serum P1NP was measured with the Rat/Mouse P1NP EIA kit (Immunodiagnostic Systems)^80^.

### RNA and protein analyses

RNA expression was analyzed by RT-qPCR. RNA was extracted with TRIZOL (Invitrogen); reverse transcribed into cDNA using an ABI High Capacity cDNA RT Kit (Invitrogen), and then analyzed using real-time quantitative PCR (SYBR Greener, Invitrogen) with gene-specific primers in triplicate. All RNA expression was normalized by ribosomal protein L19. To evaluate mTORC1 signaling in osteoclast cultures, bone marrow cells were differentiated with 40 ng/mL of mouse M-CSF (R&D Systems) in α-MEM containing 10% FBS for 3 days (days 1–3), then with 40 ng/ml of mouse M-CSF and 100 ng/mL of mouse RANKL (R&D Systems) with or without rosiglitazone (Cayman Chemical), rapamycin (LC Laboratories), or cyclosporin A (Santa Cruz) dissolved in DMSO. Cells were directly lysed in laemmli buffer (Bio-Rad) after 40–60 h of RANKL treatment. Lysates were subjected to western blot analysis with anti-p-mTOR (S2448), anti-mTOR, anti-p-S6K1 (T389), anti-S6K1 (Cell Signaling), anti-NFATc1, anti-lamin B (Santa Cruz), or anti-β-actin (Sigma) antibodies. For all immunoblot analysis, membranes were pre-cut. Protein alignments were performed by ClustalW2 and Clustal Omega.

### Cell lysis and immunoprecipitations

Immunoprecipitations were performed as previously described^81^. Briefly, bone marrow cells were differentiated with 40 ng/mL of mouse M-CSF (R&D Systems) in α-MEM containing 10% FBS for 5 days. Cells rinsed once with cold PBS were lysed in cold lysis buffer (40 mM HEPES, [pH 7.4], 1 mM NaF, 1 mM pyrophosphate, 1 mM glycerophosphate, 0.3% NP-40, and one tablet of EDTA-free protease inhibitors [Roche] per 25 mL) for NFATc1, or (40 mM HEPES [pH 7.4], 60 µM CaCl_2_, 0.3% NP-40, and one tablet of EDTA-free protease inhibitors per 25 mL) for calcineurin. The soluble fractions of cell lysates were isolated by centrifugation at 12,000 xg for 10 min at 4 °C. For immunoprecipitation, rabbit anti-mTOR (Cell Signaling) or rabbit IgG control (Santa Cruz) antibodies were added to the lysates and incubated with rotation for 1.5 h at 4 °C. A volume of 30 µL of resuspended Protein A/G Plus-Agarose (Santa Cruz) was then added, and the incubation continued for additional 1 h. Immunoprecipitates were gently washed twice with low salt wash buffer (40 mM HEPES, [pH 7.4], 100 mM NaCl, 1 mM NaF, 1 mM pyrophosphate, 1 mM glycerophosphate, 0.3% NP-40, and one tablet of EDTA-free protease inhibitors per 25 mL) for NFATc1, or (40 mM HEPES, [pH 7.4], 100 mM NaCl, 60 µM CaCl_2_, 0.3% NP-40, and one tablet of EDTA-free protease inhibitors per 25 mL) for calcineurin, and one time with cold PBS. Immunoprecipitated proteins were denatured by the addition of 20 µL of laemmli buffer (Bio-Rad) and boiling for 2 min, resolved by 12% SDS-PAGE, and analyzed by western blotting with goat anti-PP2B (calcineurin) or mouse anti-NFATc1 (Santa Cruz) antibodies. Nuclear protein extraction was previously described^82^. Briefly, adherent macrophage or osteoclast cell cultures were washed once with cold PBS, and removed with cell-lifter (Corning) in 1 mL cold PBS and pelleted by centrifugation. Cells were resuspended with Cytoplasmic Extract (CE) buffer (10 mM HEPES, 60 mM KCl, 1 mM EDTA, 0.075% NP-40, 1 mM DTT, 1 mM PMSF, and one tablet of EDTA-free protease inhibitors per 25 mL, adjusted to [pH 7.6]), and incubated on ice for 3 min. Nuclei were pelleted by centrifugation and washed once with CE buffer without NP-40. Nuclei pellets were resuspended in Nuclear Extract (NE) buffer (20 mM Tris Cl, 400 mM NaCl, 1.5 mM MgCl_2_, 0.2 mM EDTA, 1 mM PMSF, 25% glycerol, and one tablet of EDTA-free protease inhibitors per 25 mL, adjusted to [pH 8]), and incubated on ice for 30 min with intermittent vortex. Supernatants were collected after centrifugation at 12,000 rpm for 5 min, and subjected to western blot analysis.

### In vivo rapamycin treatment

Rapamycin was administered as previously described^83^. Briefly, rapamycin was dissolved in DMSO to 100 mg/mL. This stock was diluted in 5% PEG-400 / 5% Tween-20, sterile filtered, aliquoted into 1 mL portions, and stored at −80 °C. Male mice received intraperitoneally with indicated dose of rapamycin or vehicle control containing an equal volume of diluent and DMSO every other day. The abdomen was briefly swabbed with an alcohol wipe prior to injection.

### Statistical analyses

All statistical analyses were performed with Student’s *t*-test and represented as mean ± SEM unless stated otherwise. The *p* values were designated as *p < 0.05; **p < 0.01; ***p < 0.005; ****p < 0.001; and n.s., nonsignificant (*p* > 0.05).

### Data availability

All data generated or analyzed during this study are included in this published article (and its supplementary information files).

## Electronic supplementary material

Supplementary Information(PDF 4084 kb)
