# Supplementary material for: mTORC1 impedes osteoclast differentiation via calcineurin and NFATc1
Source: Commun Biol. 2018 Apr 5;1:29. doi: 10.1038/s42003-018-0028-4 (PMC6123628; doi:10.1038/s42003-018-0028-4)
Supplement: Supplementary file 1 — Supplementary Information(PDF 4084 kb) [file 42003_2018_28_MOESM1_ESM.pdf]

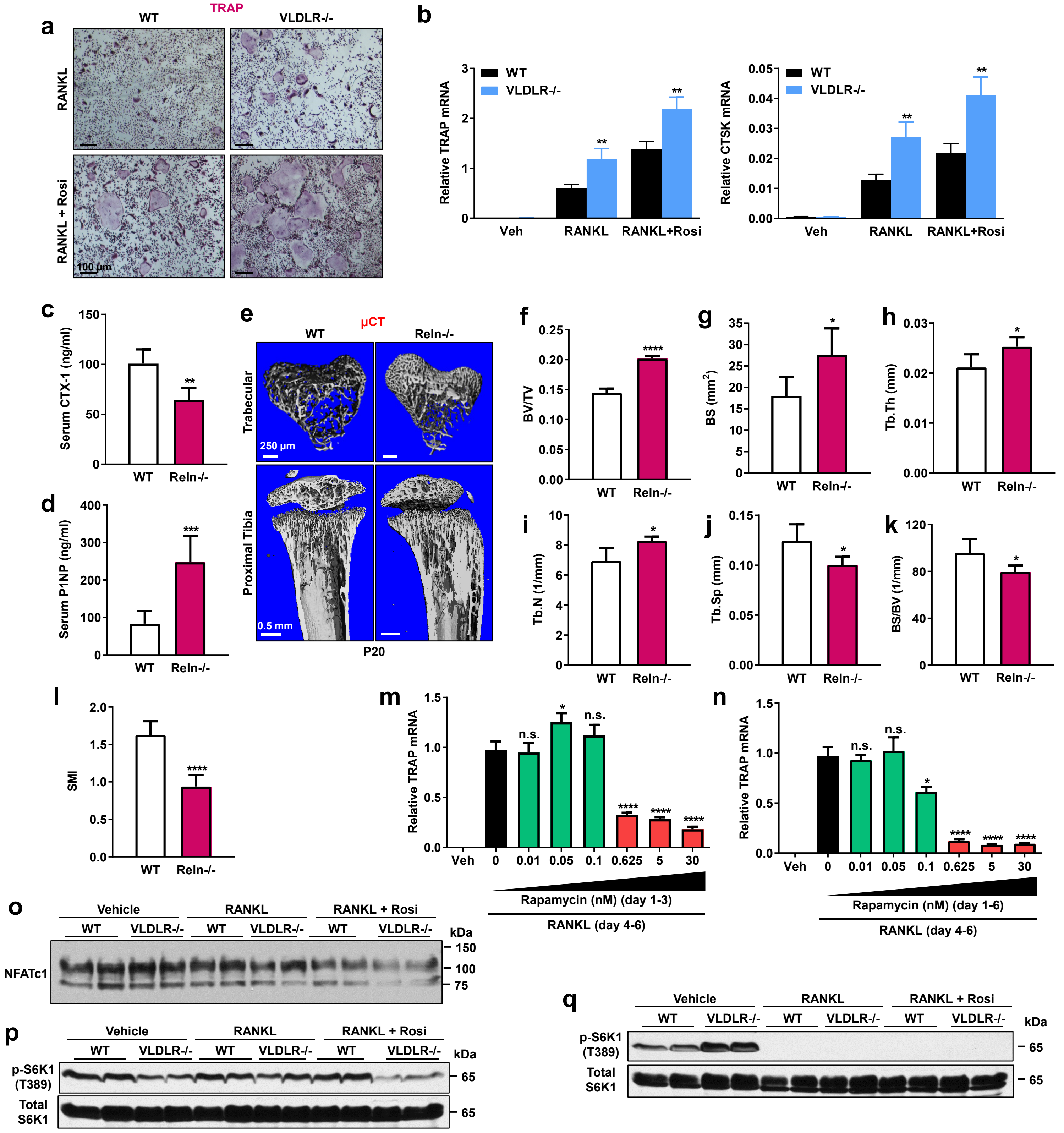

Supplementary Figure 1. Additional analyses of the relationship between mTORC1 signaling and osteoclast differentiation.
(**a**) Representative images of TRAP-stained bone marrow osteoclast differentiation cultures.
(**b**) Expression of osteoclast markers in bone marrow osteoclast differentiation cultures (n=5).
(**c**) Serum CTX-1 bone resorption marker (n=7).
(**d**) Serum P1NP bone formation marker (n=7).
(**e**) Representative µCT images of the trabecular bone of the tibial metaphysis (top) and the entire proximal tibia (bottom).
(**f-l**) Quantification of trabecular bone volume and architecture in proximal tibiae by µCT (n=7). (**f**) BV/TV, bone volume/tissue volume ratio; (**g**) BS, bone surface; (**h**) Tb.Th, trabecular thickness; (**i**) Tb.N, trabecular number; (**j**) Tb.Sp, trabecular separation; (**k**) BS/BV, bone surface/bone volume ratio; (**L**) SMI, structure model index.
(**m-n**) Expression of TRAP on day 6 of bone marrow osteoclast differentiation cultures treated with rapamycin at indicated dose and time (n=9-11).
(**o**) NFATc1 mobility shifts in bone marrow osteoclast differentiation cultures from VLDLR-/- mice or WT littermate controls 24 hr after RANKL treatment with or without rosiglitazone.
(**p**) mTORC1 signaling in bone marrow osteoclast differentiation cultures from VLDLR-/- mice or WT littermate controls 24 hr after RANKL treatment with or without rosiglitazone, measured by S6K1 phosphorylation.
(**q**) mTORC1 signaling in bone marrow osteoclast differentiation cultures from VLDLR-/- mice or WT littermate controls 72 hr after RANKL treatment with or without rosiglitazone, measured by S6K1 phosphorylation.
Error bars, SEM; *, p<0.05; **, p<0.01; ***, p<0.005; ****, p<0.001; n.s. non-significant.

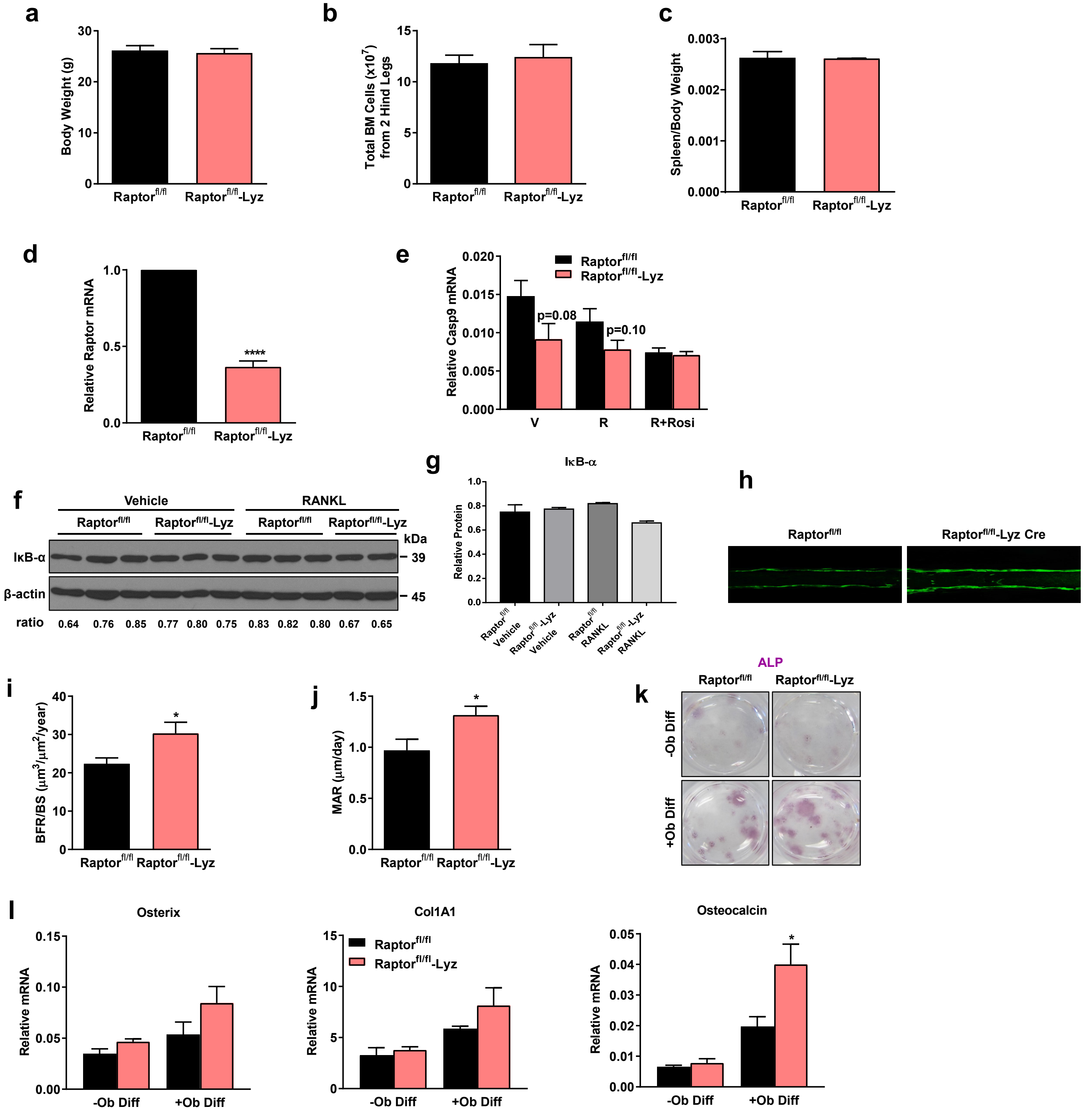

Supplementary Figure 2. Additional analyses of Raptor$^{fl/fl}$-Lyz cKO mice.
(**a**) Pup body weight at 3 months old (n=9-12).
(**b**) Total bone marrow cells in 3 months old pups (n=6).
(**c**) Quantification of spleen/body weight ratio at 6 weeks of age (n=5).
(**d**) Raptor expression in bone marrow osteoclast differentiation cultures (n=6).
(**e**) Caspase 9 expression in bone marrow osteoclast differentiation cultures (n=6).
(**f-g**) NF-κB activity measured by western blot of IκB-α at the same time point as Fig. 3f-g (50 hrs after RANKL treatment). Ratios of IκB-α/β-actin are shown for individual samples (f) and averages (g).
(**h-j**) Dynamic histomorphometry by double calcein labeling (n=6).
(**h**) Representative images.
(**i**) Bone formation rate.
(**j**) Mineral apposition rate.
(**k**) Representative images of alkaline phosphatase staining of bone marrow osteoblast differentiation cultures.
(**l**) Expression of osteoblast differentiation markers (n=6).
Error bars, SEM; *, p<0.05; **, p<0.01; ***, p<0.005; ****, p<0.001; n.s. non-significant.

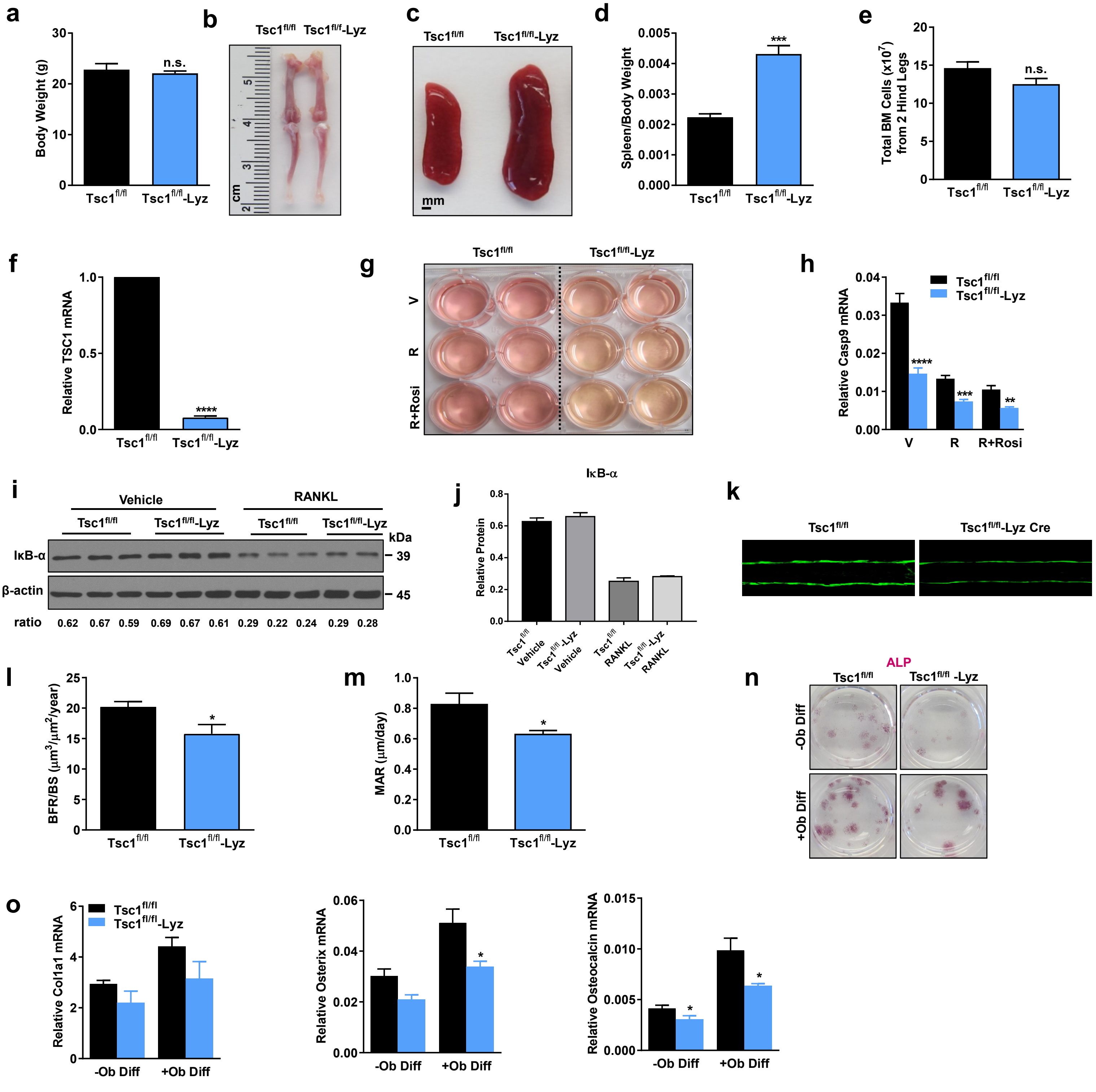

Supplementary Figure 3. Additional analyses of Tsc1fl/fl-Lyz cKO mice.

(**a**) Pup body weight at 2 months old (n=5).
(**b**) Representative image of bone length at 2 months old pups.
(**c**) Representative image of spleen at 2 months old.
(**d**) Quantification of spleen/body weight ratio (n=5).
(**e**) Total bone marrow cells in 2 months old pups (n=5).
(**f**) Tsc1 expression in bone marrow osteoclast differentiation cultures (n=6).
(**g**) Representative image of media color changes in Tsc1fl/fl-Lyz cKO bone marrow cultures.
(**h**) Caspase 9 expression in bone marrow osteoclast differentiation cultures (n=6).
(**i-j**) NF-κB activity measured by western blot of IκB-α at the same time point as Fig. 5f-g (60 hrs after RANKL treatment). Ratios of IκB-α/β-actin are shown for individual samples (i) and averages (j).
(**k-m**) Dynamic histomorphometry by double calcein labeling (n=6).
(**k**) Representative images.
(**l**) Bone formation rate.
(**m**) Mineral apposition rate.
(**n**) Representative image of alkaline phosphatase staining of bone marrow osteoblast differentiation cultures.
(**o**) Expression of osteoblast differentiation markers (n=6).
Error bars, SEM; *, p<0.05; **, p<0.01; ***, p<0.005; ****, p<0.001; n.s. non-significant.

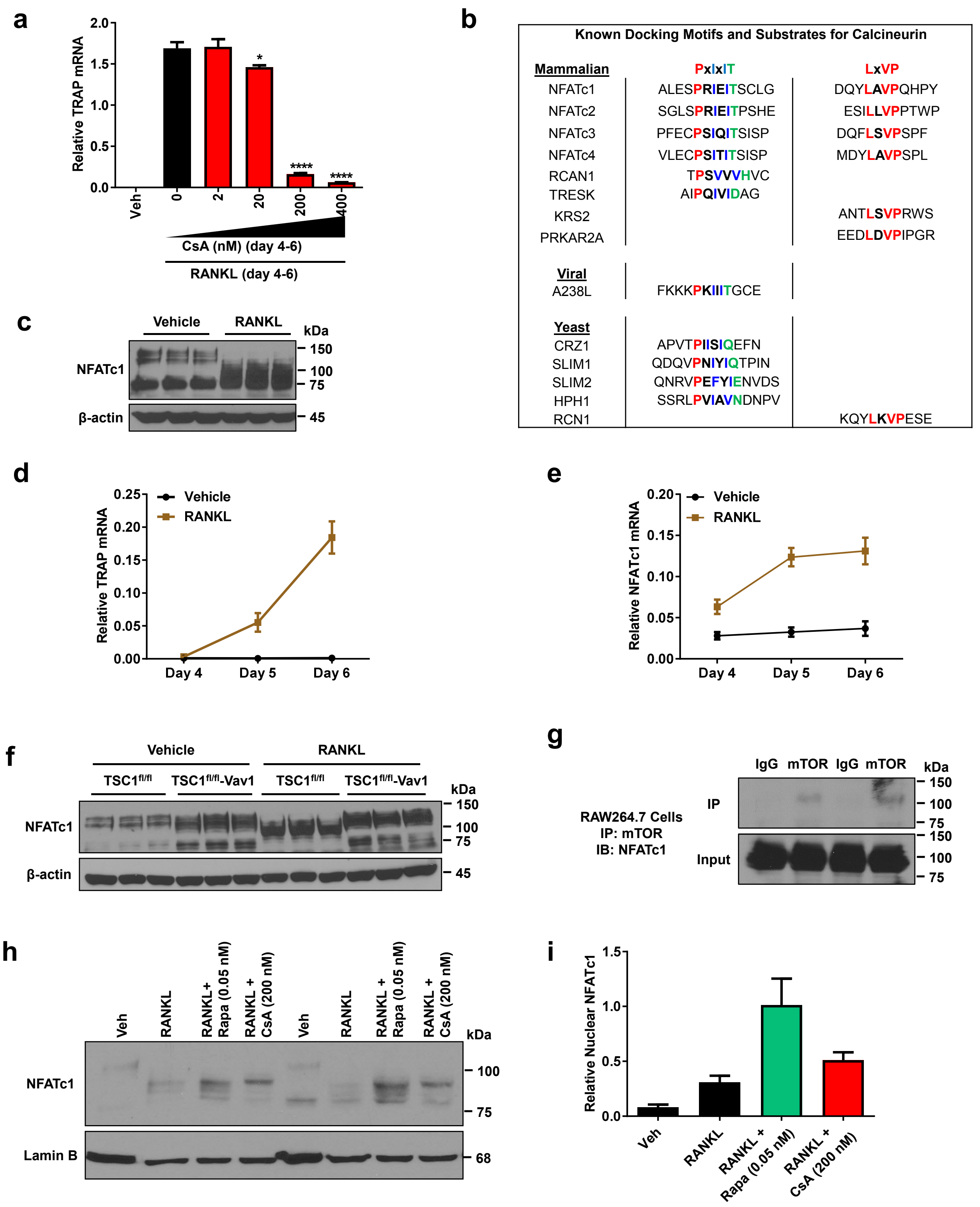

Supplementary Figure 4. Additional analyses of the calcineurin ⊣ mTORC1 ⊣ NFATc1 pathway in osteoclastogenesis.
(**a**) Expression of TRAP on day 6 in bone marrow osteoclast differentiation cultures treated with CsA at indicated dose and time (n=6).
(**b**) PxIxIT and LxVP docking motifs in known substrates for calcineurin.
(**c**) Western blot showing NFATc1 mobility shift in bone marrow osteoclast differentiation cultures 3 days after RANKL treatment.
(**d-e**) Expression of osteoclast markers (d) and osteoclastogenic transcription factors (e) in a time course of bone marrow osteoclast differentiation (n=9).
(**f**) Western blot showing NFATc1 mobility shift in bone marrow osteoclast differentiation cultures from Tsc1$^{fl/fl}$-Vav1-iCre cKO mice 60 hr after RANKL treatment.
(**g**) Western blot of NFATc1 from mTOR immunoprecipitates in RAW264.7 macrophages.
(**h**) Western blot of nuclear protein lysates from bone marrow osteoclast differentiation cultures 48 hr after RANKL treatment with or without rapamycin or CsA.
(**i**) Quantification of nuclear NFATc1/lamin B ratio (n=2).

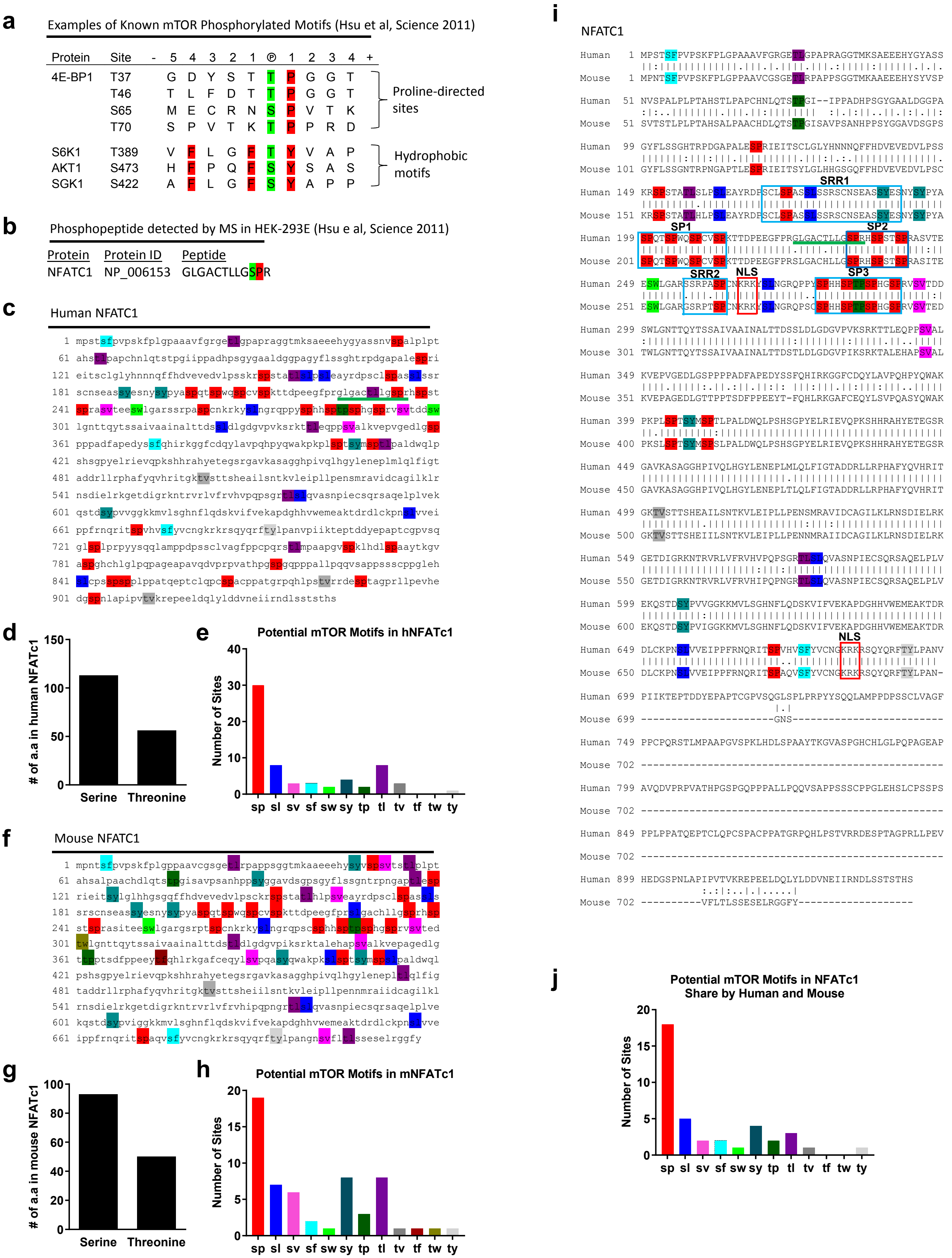
a
Examples of Known mTOR Phosphorylated Motifs (Hsu et al, Science 2011)
Protein Site - 5 4 3 2 1 ℗ 1 2 3 4 +
4E-BP1 T37 G D Y S T T P G G T
T46 T L F D T T P G G T
S65 M E C R N S P V T K
T70 S P V T K T P P R D
Proline-directed sites
S6K1 T389 V F L G F T Y V A P
AKT1 S473 H F P Q F S Y S A S
SGK1 S422 A F L G F S Y A P P
Hydrophobic motifs
b
Phosphopeptide detected by MS in HEK-293E (Hsu e al, Science 2011)
Protein Protein ID Peptide
NFATC1 NP_006153 GLGACTLLGSPR
c
Human NFATC1
d
# of a.a in human NFATc1
Serine
Threonine
e
Potential mTOR Motifs in hNFATc1
Number of Sites
sp sl sv sf sw sy tp tl tv tf tw ty
f
Mouse NFATC1
g
# of a.a in mouse NFATc1
Serine
Threonine
h
Potential mTOR Motifs in mNFATc1
Number of Sites
sp sl sv sf sw sy tp tl tv tf tw ty
i
NFATC1
Human
Mouse
SRR1
SP1
SP2
SRR2
NLS
SP3
j
Potential mTOR Motifs in NFATc1 Share by Human and Mouse
Number of Sites
sp sl sv sf sw sy tp tl tv tf tw ty

k

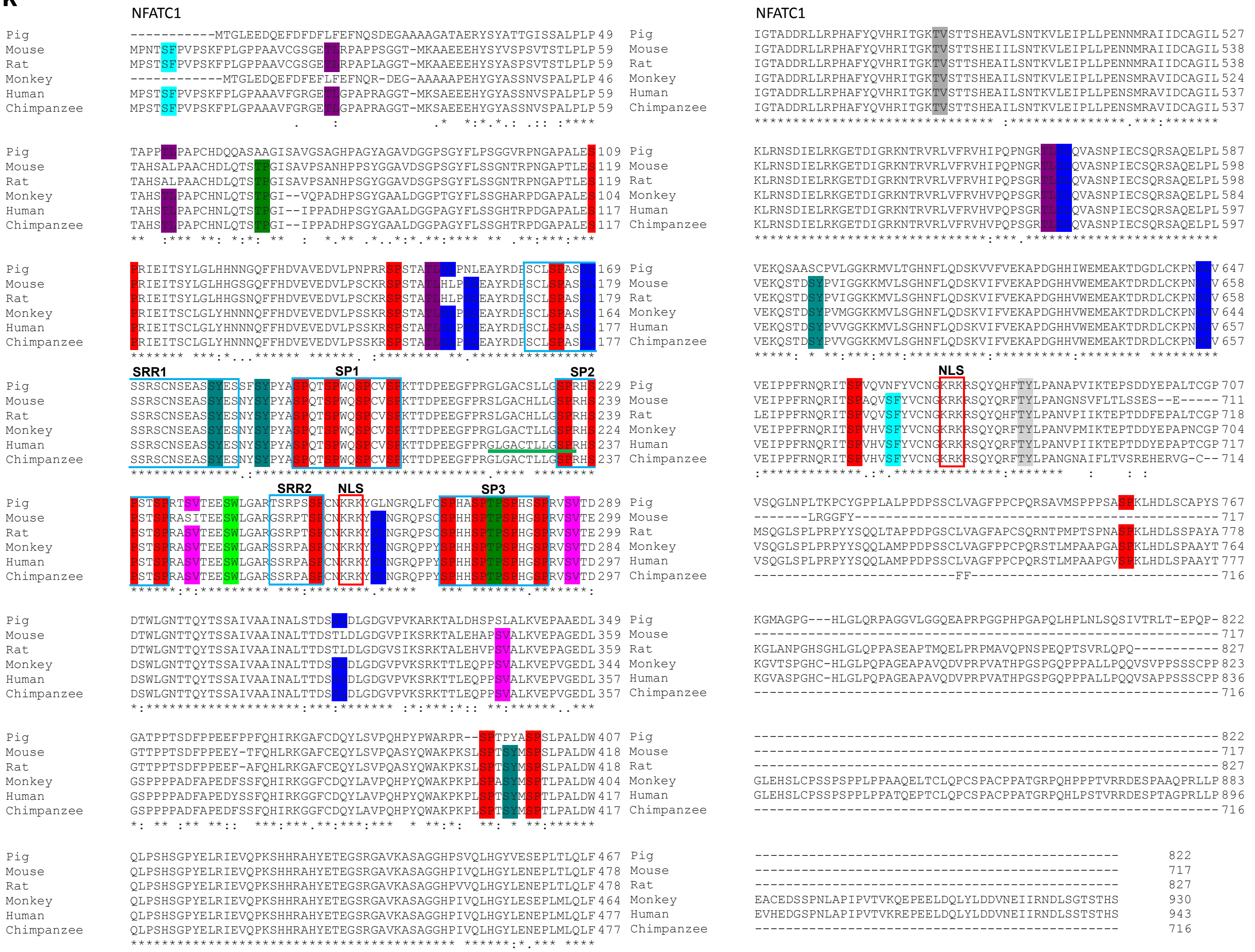

l

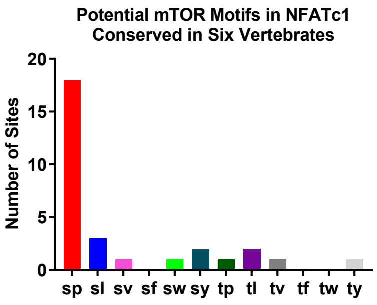

Supplementary Figure 5. Bioinformatics analysis of NFATc1.
(**a-j**) Human and mouse NFATc1.
(**a**) Examples of known mTOR phosphorylated motifs. (**b**) Phosphopeptide with serine-proline motif of NFATc1 detected in HEK-293E by mass spectrometry.
(**c-h**) Bioinformatics analysis of human NFATc1 (**c-e**) and mouse NFATc1 (**f-h**). (**c,f**) potential mTOR phosphorylation motifs colors coded as consensus sites; (**d,g**) number of serine and threonine residues; (**e,h**) quantification of consensus sites in NFATc1.
(**i**) Protein alignment of human and mouse NFATc1. SRR, serine rich region; SP, serine/proline repeats; NLS, nuclear localization signal; green underlined, phosphopeptide detected by mass spectrometry.
(**j**) quantification of consensus sites overlapping between human and mouse NFATc1.
(**k-l**) Vertebrate NFATc1 orthologs.
(**k**) Protein alignment of various NFATc1 orthologs. Domestic pig (Sus scrofa); Mouse (Mus musculus); Rat (Rattus norvegicus); Rhesus monkey (Macaca mulatta); Human (Homo sapiens); Chimpanzee (Pan troglodytes).
(**l**) quantification of consensus sites overlapping between vertebrate NFATc1 orthologs.

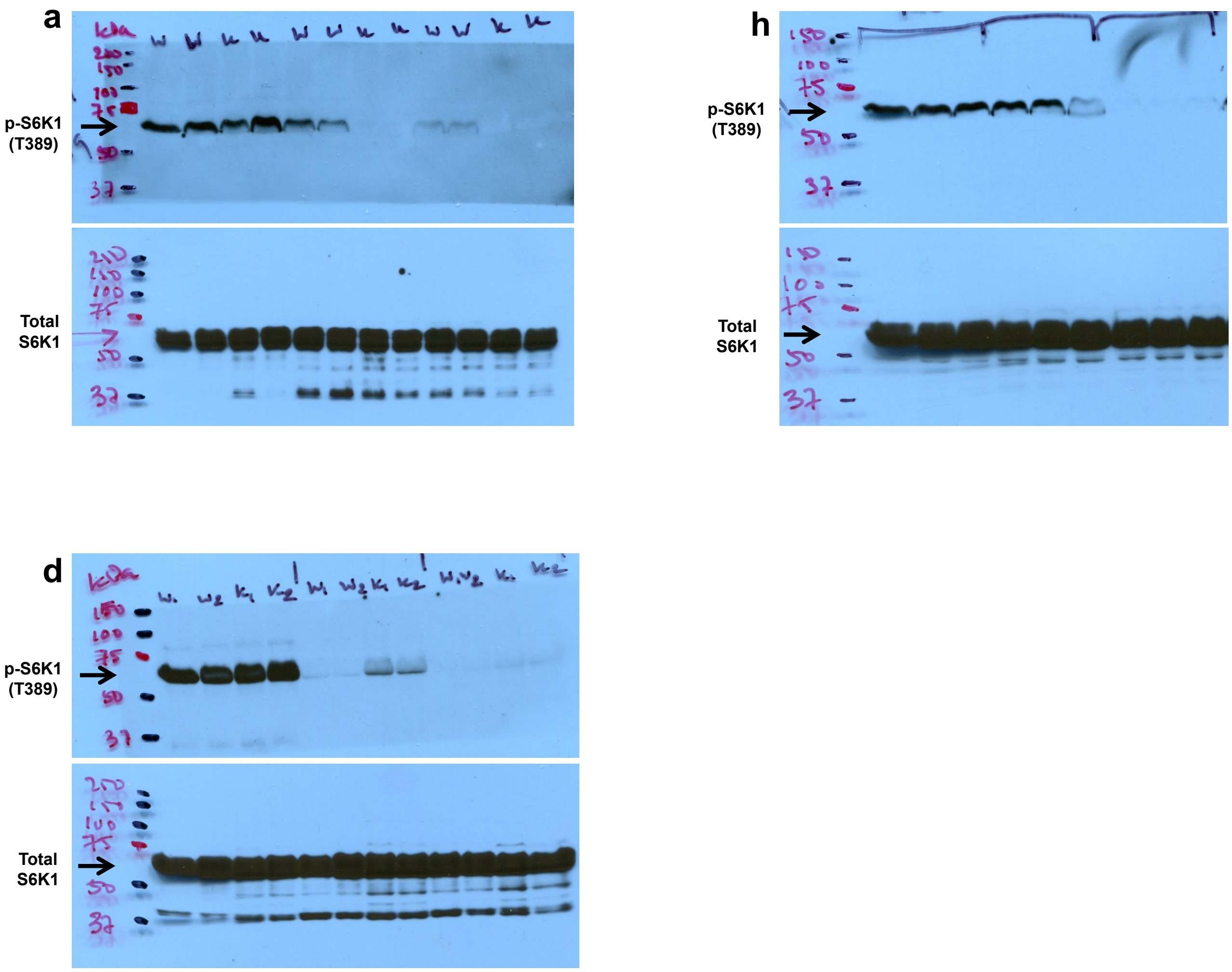

Supplementary Figure 6. Full size scans of immunoblots in Figure 1.
Panel labels correspond to panels in Figure 1.

f

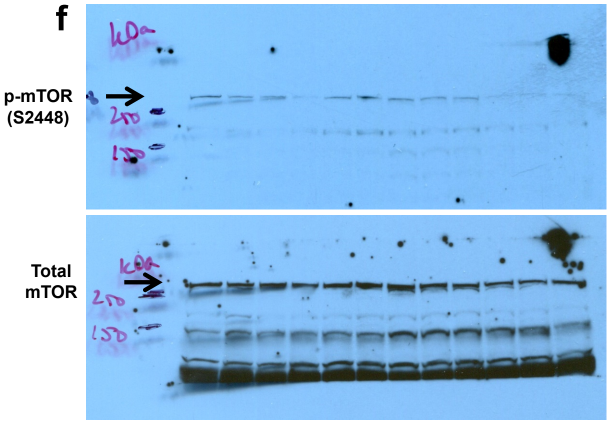

g

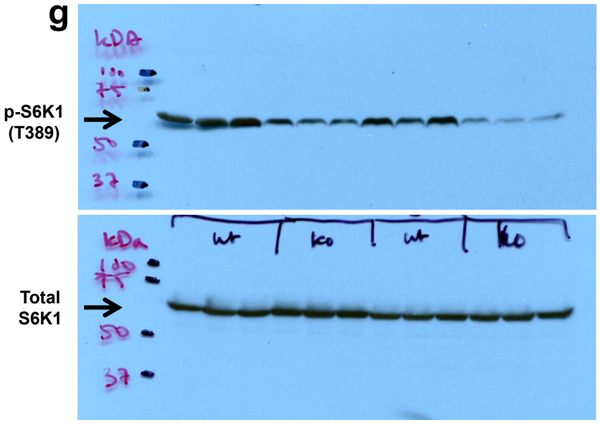

Supplementary Figure 7. Full size scans of immunoblots in Figure 3.
Panel labels correspond to panels in Figure 3.

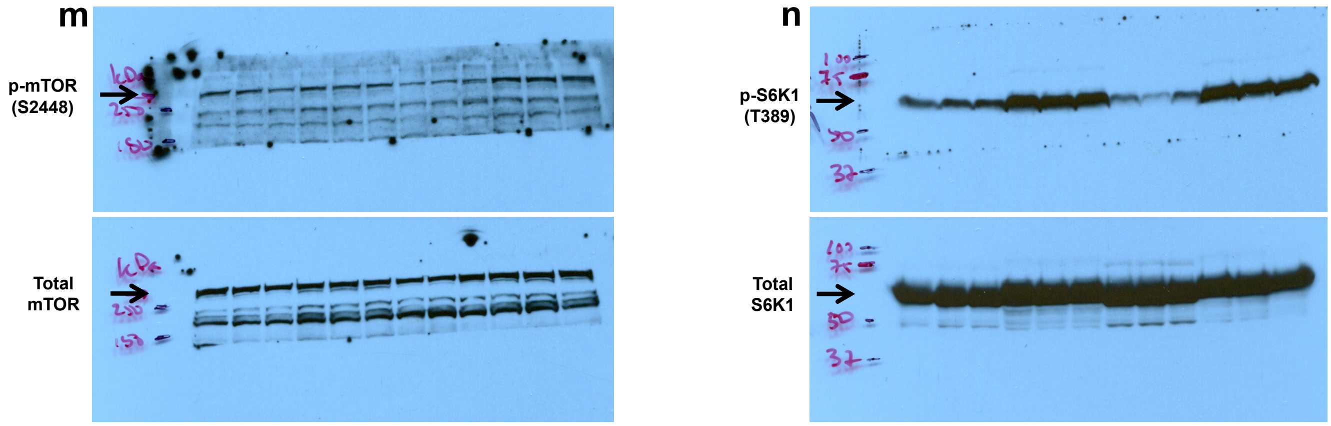

Supplementary Figure 8. Full size scans of immunoblots in Figure 4.
Panel labels correspond to panels in Figure 4.

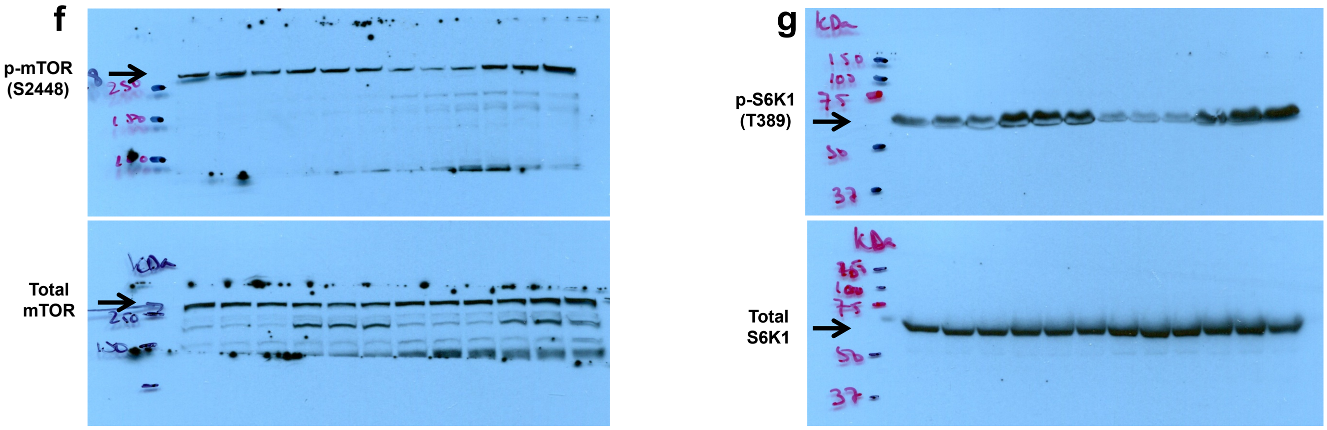

Supplementary Figure 9. Full size scans of immunoblots in Figure 5.
Panel labels correspond to panels in Figure 5.

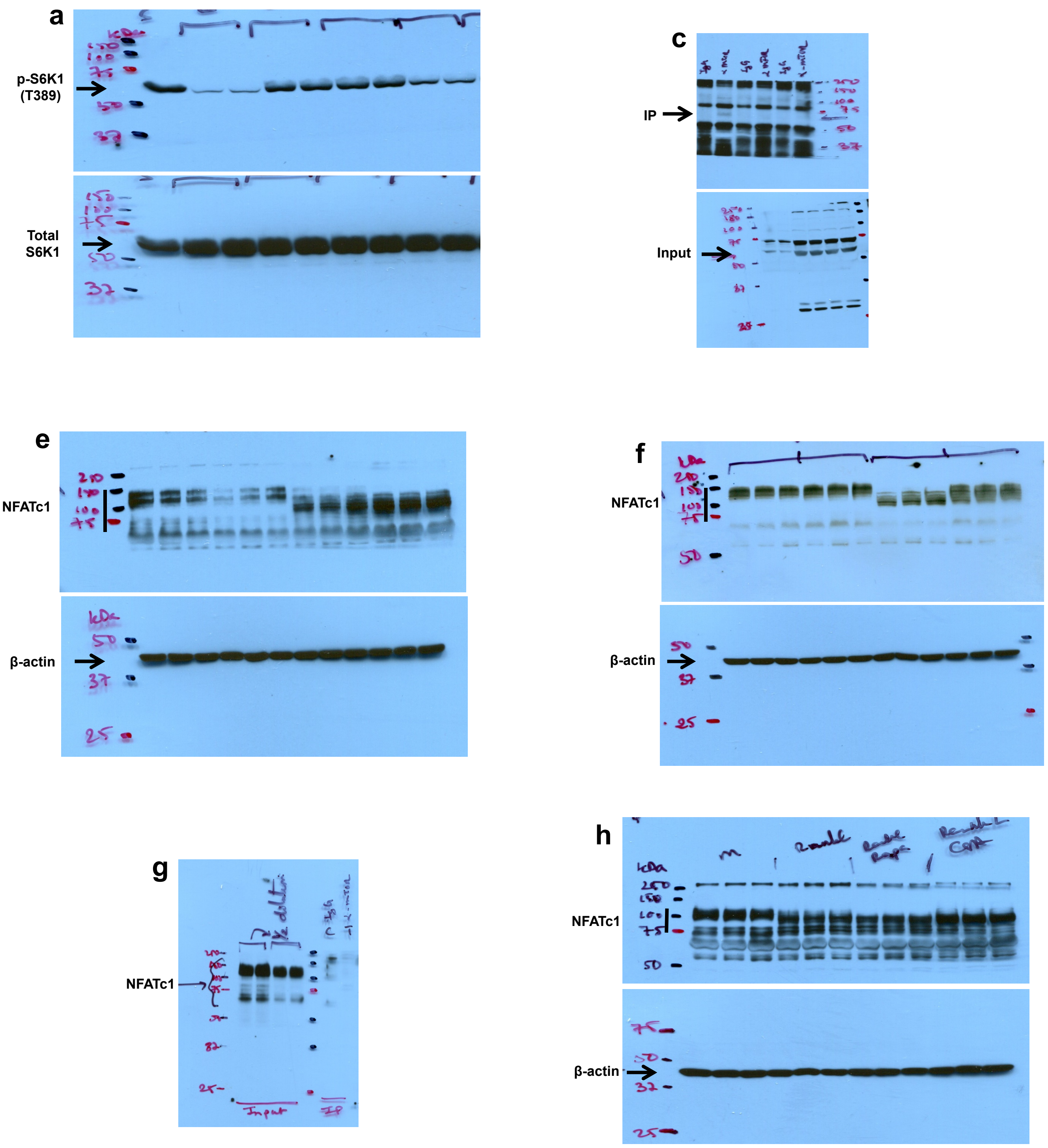

Supplementary Figure 10. Full size scans of immunoblots in Figure 6.
Panel labels correspond to panels in Figure 6.

Supplementary Table 1. Bone Marrow Cellularity of Raptor$^{fl/fl}$;Vav1-iCre Mice (n=2)

Surface Marker	Raptor$^{fl/fl}$	Raptor$^{fl/fl}$+Vav1i
Mac-1$^+$ (%)	42.20 ± 2.97	55.05 ± 3.18
Gr-1$^+$ (%)	43.05 ± 2.33	42.10 ± 2.26
B220$^+$ (%)	27.9 ± 2.0	22.8 ± 1.6
Thy1.2$^+$ (%)	1.42 ± 0.16	1.73 ± 0.24
Ter119$^+$ (%)	24.45 ± 1.06	12.35 ± 0.78

Supplementary Table 2. Bone Marrow Cellularity of Raptor$^{fl/fl}$-Lyz-Cre Mice (n=5)

Surface Marker	Raptor$^{fl/fl}$	Raptor$^{fl/fl}$-Lyz
Mac-1$^+$ (%)	45.33 ± 8.52	50.73 ± 0.72
Gr-1$^+$ (%)	44.53 ± 8.69	48.67 ± 0.32
B220$^+$ (%)	18.63 ± 3.72	17.40 ± 0.85
Thy1.2$^+$ (%)	0.68 ± 0.96	0.65 ± 0.98
Ter119$^+$ (%)	32.0 ± 6.24	27.23 ± 2.36

Supplementary Table 3. Bone Marrow Cellularity of Tsc1$^{fl/fl}$-Vav1-iCre Mice (n=3)

Surface Marker	Tsc1$^{fl/fl}$	Tsc1$^{fl/fl}$-Vav1i
Mac-1$^+$ (%)	35.81 ± 11.61	63.73 ± 7.64*
Gr-1$^+$ (%)	35.56 ± 11.17	61.50 ± 7.61*
B220$^+$ (%)	27.43 ± 2.82	14.33 ± 3.94**
Thy1.2$^+$ (%)	1.37 ± 0.68	1.99 ± 1.19
Ter119$^+$ (%)	28.77 ± 6.33	14.97± 4.12*

Supplementary Table 4. Bone Marrow Cellularity of Tsc1$^{fl/fl}$-Lyz-Cre Mice (n=5)

Surface Marker	Tsc1$^{fl/fl}$	Tsc1$^{fl/fl}$-Lyz
Mac-1$^+$ (%)	36.203 ± 4.47	39.22 ± 5.75
Gr-1$^+$ (%)	37.07 ± 5.08	38.88 ± 5.67
B220$^+$ (%)	25.50 ± 4.09	29.34 ± 2.08
Thy1.2$^+$ (%)	1.75 ± 0.24	1.34 ± 0.15*
Ter119$^+$ (%)	34.47 ± 4.10	26.40 ± 3.39**
